# Building a construct-valid battery of performance and self-report indicators of sustained attention consistency

**DOI:** 10.3758/s13428-025-02798-w

**Published:** 2025-10-08

**Authors:** Matthew S. Welhaf, Matt E. Meier, Michael J. Kane

**Affiliations:** 1 Appalachian State University, Boone, NC USA; 2 UNC Greensboro, Greensboro, NC USA

**Keywords:** Attention consistency, Mind wandering, Reaction time variability, Construct validity

## Abstract

Previous work has argued that the ability to sustain attention consistency can be best modeled as the individual-difference covariation in objective performance-based measures (e.g., reaction-time [RT] variability; accuracy) and self-report measures of task-unrelated thought (TUT). Latent variable studies demonstrate that a general, higher-order attention consistency factor correlates more strongly with nomological network constructs than do either lower-order, measurement-specific factors. The present study aimed to replicate and extend this measurement approach by building a construct-valid battery of sustained attention consistency tasks and testing associations with the conative factors of task interest and success motivation. We analyzed data from 402 subjects who completed a battery of seven attention-consistency functions and found that the hierarchical model provided an adequate fit to the data. Further, attention-consistency associations with motivation and interest, while evident with the lower-order factors, were again stronger with the general higher-order factor (and each conative factor predicted unique variance in general attention consistency in structural regression models). We also refined our task battery by removing poor-performing indicators and demonstrated similar patterns of correlations among the attention and conative factors. We suggest that studies examining attention consistency should use a combination of performance and self-report indicators to capture its individual-differences variation in the most construct valid way. We finally provide recommendations on which tasks and measures might be most useful when measuring sustained attention consistency in future research.

**Supplementary Information:**

The online version contains supplementary material available at 10.3758/s13428-025-02798-w.

## Introduction

Sustaining consistent attentional focus is critical to many everyday actions, including driving (Burdett et al., 2019; Yanko & Spalek, 2014), academic learning (Hollis & Was, 2016; Kane et al., 2021a, 2021b; Unsworth & McMillan, 2017), and job performance (Dane, 2018; Ibaceta et al., 2024; Li, 2023). Failing to maintain attention consistently may result in errors ranging from merely annoying to significantly costly to catastrophic. It is therefore important to accurately assess and account for individual differences in sustained attention consistency (Unsworth & Miller, 2021).

We have recently suggested that the ability to sustain attention consistency is best reflected by the individual-differences overlap in “objective” (performance-based) and “subjective” (self-report-based) measures, rather than by either measurement approach alone (Welhaf & Kane, 2024a, 2024b). The current study aims to further assess the construct validity of attention consistency measures by using an a priori set of tasks and measures that allow for a more comprehensive assessment of the construct. Additionally, we investigate how individual differences in facets of motivation predict the ability to maintain consistent attention from moment to moment.

## Measuring sustained attention consistency

Our view (Welhaf & Kane, 2024a, 2024b) argues that sustained attention consistency can be best measured by the covariation in task-performance *and* momentary-self-report measures. In our prior work, we extensively reviewed these approaches and their limitations when used in isolation (Welhaf & Kane, 2024b), so we only briefly discuss them here.

## Performance-based measures

Successful attention consistency should manifest as steady task performance across trials and time. Much of the literature on attention lapses has focused on measures of reaction time (RT)—including RT variability (e.g., intra-individual RT standard deviation across trials), rare but extremely slow RTs (e.g., lapses), or model parameters derived from participants’ complete RT distribution (e.g., from ex-Gaussian or drift diffusion models)—that might closely reflect momentary lapses. However, using only RT-based variables to assess attention consistency provides a suboptimal measure. Individual variation in RT-based measures of attention consistency might also be due, in part, to non-attention-consistency processes. For example, momentary shifts in a participant’s RTs could arise from speed–accuracy trade-offs, unintentional bodily actions like sneezing or blinking, or even changes in local task parameters (e.g., mixed incongruent and congruent trial types in a Stroop task).

Successful attention consistency should also result in more accurate task performance. For example, Cheyne et al. (2009) proposed that different errors in a go/no-go task (e.g., omission versus commission errors) might reflect distinct, yet related, forms of attentional disengagement, beyond what is captured by variability in RT during the task. Several studies have found moderate-to-strong correlations between RT variability and accuracy scores in varied sustained attention tasks. In the sustained attention to response task (SART), for example, intra-individual RT variability and dʹ (a signal detection measure of discrimination) correlate at least moderately with each other (*r*s ≈.25–30; e.g., Banks & Welhaf, 2022; Kane et al., 2021a, 2021b). Recent work using the gradual-onset continuous performance task (gradCPT), a variant of the SART, has shown strong correlations between RT variability and accuracy measures (*r* =.76; Fortenbaugh et al., 2015). Fortenbaugh et al. (2015) showed that RT variability and dʹ formed a coherent “sustained attention ability” factor, which showed distinct age-related changes compared to a “sustained attention strategy” factor that was made up of mean RT and signal detection bias. Following such findings, a primary goal of the current study was to broaden the assessment of attention consistency by incorporating tasks that provide accuracy-based, in addition to RT-based measures, while also using tasks that present a variety of stimuli and task demands.

## Momentary self-report (thought sampling) measures

While failures of attention consistency can manifest in poorer behavioral outcomes, like RT variability or task errors, another common way to experience and assess attention inconsistency is via the subjective experience of mind wandering. Several researchers have used the thought probe technique to capture participants’ ongoing thoughts during different task contexts (for a review, see Weinstein, 2018). In this procedure, participants are randomly interrupted and asked to report whether their immediately preceding thoughts were on- or off-task. Task-unrelated thoughts (TUTs)—one variety of mind-wandering—have been shown to predict poorer ongoing task performance (e.g.,Brosowsky et al., 2023; McVay & Kane, 2009; Seli et al., 2013; Van Vugt & Broers, 2016) and are related to several key individual-differences measures, such as working memory capacity and attention control ability (e.g.Banks & Welhaf, 2022; Barrington & Miller, 2023; Kane et al., 2016; McVay & Kane, 2012b; Rummel & Boywitt, 2014; Soemer & Schiefele, 2020; Unsworth et al., 2021).

Mind-wandering can vary along many dimensions (Seli et al., 2018b, 2018c), including varieties of thought content (see Welhaf et al., 2020). For example, TUT assessments have focused on their temporal orientation (Baird et al., 2011; Maillet et al., 2017; McVay et al., 2013; Stawarczyk et al., 2013), emotional valence (Banks & Welhaf, 2022; Banks et al., 2016; Spronken et al., 2016; Taruffi, 2021; Tusche et al., 2014), self vs. other orientation (Huijser et al., 2018; Kanske et al., 2016; Lux et al., 2022), internal vs. external orientation (Robison & Unsworth, 2015; Unsworth & McMillan, 2014; Zhang et al., 2023), intentionality (O’Neill et al., 2021; Seli et al., 2016, 2017, 2018a, 2018b, 2018c), and categories of thought content, such as worries and fantastical daydreams (Kane et al., 2021a, 2021b; McVay & Kane, 2009, 2012a, 2013). However, individual studies of mind wandering typically focus on just a single dimension of TUTs, based on the research question (for a review, see Weinstein, 2018). It is therefore unclear whether these different dimensions of mind wandering capture slightly different sources of individual-differences variance. If they do, then probing participants about different aspects of their mind wandering might validly diversify assessments of attention consistency by eliminating mono-operation bias. Thus, a primary goal of the current study was to also broaden TUT assessment by asking participants to report on two different aspects of mind-wandering across different tasks.

## Current evidence for the construct validity of attention consistency

Welhaf and Kane (2024b) tested the construct validity of the individual-differences covariation between performance and thought-report measures of attention consistency. In a reanalysis of two large-scale latent variable studies (Kane et al., 2016; Unsworth et al., 2021), we showed that task-performance and TUT-rate factors were positively correlated, with *r*s =.30 to.40. Participants with a greater propensity to mind-wander during simple laboratory tasks also tended to show greater RT variability and lower accuracy. Critically, we also demonstrated that a hierarchical factor model can effectively represent the covariation in individual differences between performance-based and thought-report measures of attention consistency.

The strongest evidence for the construct validity of this approach to measuring attention consistency came from assessing the general attention consistency factor’s correlations with the other constructs measured in the reanalyzed studies. Several traits and cognitive abilities, including working memory capacity, processing speed, Big-5 personality factors, self-reported cognitive failures, and self-reported symptoms of positive schizotypy, all correlated weakly to moderately with the lower-order performance and thought-report factors. However, correlations with the general higher-order factor were as strong as, if not stronger than, those with either method-specific factor on their own. From this, Welhaf and Kane (2024b) concluded that measurement error specific to the lower-order factors contributed to weaker observed relationships between the constructs. Using the shared variance between the performance and self-report first-order factors provided a more construct-valid assessment of sustained attention consistency (see Zanesco et al., 2024 for a similar approach in examining the covariance between mind wandering and the vigilance decrement).

## Attention consistency and motivation

Welhaf and Kane (2024b) hypothesized that individual differences in sustained attention consistency would be substantially correlated with contextual or conative factors, like motivation and task interest (e.g., Robison & Unsworth, 2018; Unsworth & McMillan, 2013; Unsworth & Miller, 2024), and that these should be more strongly correlated with the general attention consistency factor than with either the performance-specific or thought-report-specific factors. That is, participants who report being more highly motivated or interested in an ongoing task should be better able (or more inclined) to sustain attention across the task, yielding more accurate and consistent performance and more on-task thinking. Previous correlational and experimental work supported this claim: Individuals who self-report being more motivated or interested typically show better performance on indicators of attention consistency (Esterman et al., 2014; Massar et al., 2016; Strayer et al., 2024; Unsworth et al., 2022a, 2022b). Likewise, higher levels of self-reported motivation or interest consistently predict fewer instances of TUTs (Barrington & Miller, 2023; Brosowsky et al., 2023; Kane et al., 2017; Kawagoe et al., 2020; Robison & Unsworth, 2018; Unsworth & McMillan, 2013). Thus, individual differences in conative factors should correlate with general sustained attention consistency ability.

We found, however, that the general sustained attention consistency factor was *perfectly* correlated with several contextual factors. More precisely, the factor-analytic model did not adequately fit the data, and inspection of the model summary indicated that the motivation factor, among several others, correlated with attention consistency with *r* > 1.0. As discussed in Welhaf and Kane (2024b), we do not think attention consistency is isomorphic with motivation or interest for several interrelated reasons concerning measurement error.

In the re-analyzed data set (Unsworth et al., 2021), these conative constructs were assessed using a single-item measure at the end of the relevant task. Although such measures may have low reliability, motivation ratings correlated significantly across tasks in this dataset. Perhaps more problematic, then, are the following concerns. First, the single-item motivation questions were always answered after the task was completed, so challenges in sustaining attentional focus during the task may have affected participants’ inferences about their motivation. Second, single-item measures, regardless of when they are completed, may not adequately capture variance in multifaceted constructs like motivation (Allen et al., 2022; for a review, see Touré‐Tillery & Fishbach, 2014). Using a single item asking, “*How motivated were you to perform the task?*” might have different meanings for different participants. For example, motivation could be viewed as outcome-focused versus process-focused (Touré‐Tillery & Fishbach, 2011). Outcome-focused motivation describes the action of “*getting the task done*,” whereas process-focused motivation reflects more on the internal benefits of doing the task and pursuing the goal and less on goal completion. Relying on a single-item measure may not adequately capture what it means to be motivated during a task and this, in turn, may provoke participants to rely on folk theories of motivation and performance, such that sensed attention difficulties might affect motivation ratings.

The Dundee State Stress Questionnaire (Matthews, 2021; Matthews et al., 2002) provides a nuanced and multidimensional set of questions about task motivation. The original scale developed and validated by Matthews et al. (2002) consisted of a “Task Engagement” factor comprised of four scales: energetic arousal, task interest, success motivation, and concentration. Two of these scales, task interest and success motivation, particularly capture the domain of motivation (Matthews, 2016). Several studies have shown that this task engagement factor is related to performance in traditional long-duration vigilance tasks, visual search tasks, and complex real-world tasks (for a review, see Matthews, 2021). Thus, the present study used a broader motivation assessment to better examine the relationship between attention consistency and participants’ conative states.

## The current study

The present study expanded on our previous construct validation work by examining the factor structure and nomothetic network of sustained attention consistency with an a priori set of tasks and measures that all had some reliance on sustained attention consistency. That is, we do not suggest that the selected tasks exclusively measure sustained attention consistency (as no measure is process-pure). Instead, we selected these tasks in hopes of building a battery from a diverse combination of tasks and measures that broadly reflect the attention consistency construct. Welhaf and Kane (2024b) relied on existing datasets that included tasks that were not optimally designed to assess sustained attention consistency, and so had the following limitations: (a) The dependent measures we derived for each task’s performance were almost exclusively based on RT. Thus, individual variation in these performance indicators of attention consistency could have also been influenced by other processes, like speed–accuracy trade-offs or changes in response strategies across the task, and (b) The task stimuli were very similar, in that they were simple (e.g., words, arrows, numbers) and presented visually.

For the present study, we selected tasks that not only required sustained attention consistency for successful performance but that also varied in their stimuli, response requirements, and other demands unrelated to sustained attention (see Table 1 of Welhaf & Kane, 2024b, for examples). We did this to ensure that the shared variance among our performance indicators would not be confounded by specific processes or abilities unrelated to attention consistency, thereby avoiding mono-method bias (e.g., Shadish et al., 2002). For example, some of our tasks presented either visually degraded or highly similar visual stimuli, which should challenge participants’ visual discrimination ability (e.g., Degraded Vigilance Task; Continuous Temporal Expectation Task). Other tasks presented auditory, rather than visual, stimuli and required maintaining a consistent response rhythm (e.g., Metronome Response Task; Finger Tapping Task). Traditional attention measures present mundane and uninteresting stimuli, resulting in repetitive and boring tasks that may be engaged in with little effort, at least for some participants (DeRight & Jorgensen, 2015). One way to improve engagement, without sacrificing validity, is to gamify tasks by presenting more real-life scenarios or interesting stimuli (Lumsden et al., 2016). To this end, we adapted two traditional cognitive tasks (AX-Continuous Performance Task; Psychomotor Vigilance Task) to promote engagement and measure sustained attention consistency under somewhat more arousing conditions. Finally, we used some tasks with RT-based dependent measures and others with accuracy-based measures (and some with both).

**Table 1:** Descriptive statistics

	*M*	*SD*	Min	Max	Skew	Kurtosis	*n*
SART RTsd	141.18	47.59	26.60	313.33	0.72	1.08	378
PVT Slowest 20%	547.95	123.88	324.07	955.17	0.73	0.10	381
MRT RRT	8.03	0.65	6.56	10.48	0.36	0.23	379
MotPVT Slowest 20%	463.19	81.24	300.15	763.42	0.91	0.95	398
AX-CPT dʹ	1.94	1.20	– 2.97	3.94	– 0.67	0.64	234
DVG dʹ	2.63	1.01	– 0.70	4.98	– 0.63	0.50	401
SART Omissions	0.09	0.08	0.00	0.30	0.87	– 0.24	378
CTET Block 1 dʹ	3.80	0.71	0.76	4.72	– 0.91	1.30	380
CTET Block 2 dʹ	3.61	0.78	0.76	4.72	– 0.82	0.81	380
SART TUT	0.40	0.24	0.00	1.00	0.31	– 0.59	378
PVT TUT	0.55	0.27	0.00	1.00	– 0.24	– 0.78	381
MRT TUT	0.60	0.28	0.00	1.00	– 0.60	– 0.47	379
MotPVT TUT	0.31	0.28	0.00	1.00	0.70	– 0.53	398
AX-CPT Pre-DSSQ	24.61	3.40	14.00	32.00	– 0.56	0.46	233
AX-CPT Post-DSSQ	24.05	4.59	7.00	34.00	– 0.54	0.41	234
SART Pre-DSSQ	22.72	4.30	7.00	32.00	– 0.46	0.36	378
SART Post-DSSQ	20.36	5.44	7.00	35.00	– 0.18	– 0.35	378
PVT Pre-DSSQ	23.74	4.73	10.00	35.00	– 0.17	– 0.33	381
PVT Post-DSSQ	21.55	5.71	7.00	35.00	– 0.05	– 0.52	381
MotPVT Pre-DSSQ	24.49	4.63	11.00	35.00	– 0.26	– 0.13	398
MotPVT Post-DSSQ	23.97	5.58	7.00	35.00	– 0.27	– 0.51	398

SART = Sustained Attention to Response Task; PVT = Psychomotor Vigilance Task; MRT = Metronome Response Task; MotPVT = Motivation PVT; DVG = Degraded Vigilance Task; CTET = Continuous Temporal Expectancy Task; RTsd = Intra-individual RT standard deviation; Slowest 20% = Mean RT of longest 20% of RTs; RRT = Rhythmic Response Time; TUT = proportion of task-unrelated thoughts in task; DSSQ = Dundee State Stress Questionnaire raw item total score

We also extended our self-report, thought-sampling indicators of attention consistency by using two different thought probe menus across different tasks. TUT assessments in individual differences studies typically ask participants to report on their thoughts using the same response options across all probed tasks, which might contribute method-specific variance. That is, some participants might be consistently biased against reporting some kinds of thoughts, or some participants might consistently interpret a response option peculiarly. To reduce such measurement error across the task battery, each probed task presented one of two thought-content-based probe types, each used in prior research. One probe type asked about prototypical thought categories during ongoing tasks (e.g., Kane et al., 2016; McVay & Kane, 2012a) and one asked about the temporal orientation of thoughts (e.g., McVay & Kane, 2012b; Unsworth & McMillan, 2013). Content-based probes like these are commonly used and yield reliable and valid thought reports (e.g., Kane et al., 2016, 2021a, 2021b).

We had two main questions. First, can we extend our prior findings regarding the lower-order performance factor and TUT-rate factor (Welhaf & Kane, 2024b) to account for different dependent measures (i.e., accuracy versus RT variability; different probes for TUT rate assessments) that reflect the ability to sustain attention consistency, at least in part? Second, can we provide further evidence regarding the construct validity of our measurement approach by examining the association between a general factor of sustained attention consistency (spanning performance and self-report assessments) and two facets of motivation, success motivation, and task interest?

## Methods

### Participants

We recruited participants from the undergraduate research pools at UNC Greensboro (UNCG) and Western Carolina University (WCU). Data collection at UNCG began in the Spring of 2020 but was postponed due to the onset of the COVID-19 pandemic. Data collection began again at UNCG and began at WCU in the Spring of 2023, and continued at UNCG through Fall 2023 and into the first few weeks of the Spring 2024 semester. Data collection at WCU ended after the Spring 2023 semester. A total of 429 undergraduates consented to participate for partial credit toward a course requirement (UNCG *N* = 357; WCU *N* = 72). After data exclusions, described below, all analyses were conducted on a sample of 402 participants, which is large enough to provide stable estimates of correlations (Schönbrodt & Perugini, 2013), and which is between the sample sizes of the studies re-analyzed by Welhaf and Kane (2024b), with *N*s = 358 (Unsworth et al., 2021) and 545 (Kane et al., 2016).

### Procedure

Participants completed eight tasks in a 2-h session in groups of one to six, after first providing informed consent. Task order was fixed and proceeded as listed in the “Tasks and Measures” Section. An experimenter remained present throughout the session and read aloud all task instructions. The study protocol was reviewed and approved by the Institutional Review Boards at UNCG and WCU.

### Tasks and measures

#### Performance-based attention consistency measures

As discussed above, we selected tasks to represent a variety of stimuli, non-sustained-attention task demands, and performance indicators, and thereby to minimize mono-method bias and measurement error. This strategy parallels our approach to using more than one type of content-based thought probe across tasks (discussed below). The dependent measures selected here are commonly used in the literature for these tasks and provide a balance of accuracy- and RT-based measures across (and in some cases within) tasks.

*Avengers AX-CPT (adapted from Braver et al., 2005; Redick, 2014).* The stimuli in this task consisted of illustrated portrait images of Avengers characters from the Marvel Cinematic Universe (see https://marvelcinematicuniverse.fandom.com/wiki/Category:The_Avengers_Characters; see also Supplemental Materials). As in standard AX-CPT tasks, trials were constructed as cue-probe pairs.

Each trial presented a cue image for 500 ms, a blank screen for 400 ms, a second (probe) image for 500 ms, and a blank screen for 200 ms (total trial duration = 1600 ms). We instructed participants to respond to each image by pressing a key. Specifically, they should press the 1 key on the numeric keypad for Captain America if—and only if—he was preceded by Iron Man (the AX trial type), but to press the 2 key on the numeric keypad for any other Avengers character (e.g., Iron Man, Hawkeye, Black Widow) and any appearance of Captain America preceded by a different Avengers character.

Participants completed 18 practice trials. After the practice trials, participants completed two seamless blocks of 36 cue-probe trials (for a total of 72 cue-probe trials), of which 72% were AX trials (Captain America preceded by Iron Man), 11% were AY trials (e.g., Hawkeye preceded by Iron Man), 11% were BX trials (e.g., Captain America preceded by Black Widow), and 6% BY trials (e.g., Hawkeye preceded by Black Widow). The dependent measure was a signal detection measure of accuracy (dʹ) using AX hits and BX false alarms. The task took approximately 10 min to complete.

*Degraded Vigilance Task (DVG; *Temple et al., 2000; Unsworth et al., 2009*)*. Participants responded by pressing the 0 key any time a target stimulus (“0”) appeared centrally onscreen, while ignoring central appearances of non-target stimuli (“D” or backward “D”); all targets and non-targets appeared against a background “wallpaper” of repeating Os. Target and non-target stimuli appeared very briefly (40 ms) before disappearing. Participants completed 12 practice trials before 360 experimental trials, of which 60 were target 0 s. We used dʹ as the dependent measure. The task took approximately 10 min to complete.

*Semantic Sustained Attention to Response Task (SART; *McVay & Kane, 2012a, 2012b*).* This go/no-go task required participants to press the space bar for words from one category (animals; 89% of trials) and to withhold responses to another category (vegetables; 11% of trials). Participants practiced the task for ten trials by pressing the space bar whenever they saw a boy’s name and withholding responses to girls’ names. The non-practice trials of the SART began with ten unanalyzed buffer trials. Participants completed 480 critical trials across four seamless blocks, each comprising three seamless mini-blocks. Each mini-block presented 40 trials, with 36 “go” (non-target) trials and four “no-go” (target) trials, presented in a different random order for each participant. The dependent measures for the SART were omission errors (i.e., “go” trial error rates) and the intrasubject standard deviation (SD) of RTs to “go” (animal) trials (Banks & Welhaf, 2022; Kane et al., 2016, 2021b; Robison et al., 2023). The task took approximately 20 min to complete.

*Psychomotor Vigilance Task (PVT; *Dinges & Powell, 1985*).* Each trial began with a set of blue zeros (“00.000”) in the center of a white screen. After an unpredictable interval from 1–10 s (in 1000-ms increments), the zeros began counting upward in milliseconds. Participants were instructed to stop the counter by pressing the spacebar as quickly as possible, after which the zeros turned red to provide RT feedback. If the spacebar was mistakenly pressed before the timer started (resulting in a false alarm) that trial was repeated at the end of the task.

Participant completed two seamless blocks of 45 trials, for a total of 90 trials. In each block, participants completed four trials at each interstimulus interval (ISI). Additionally, five trials per block served as'yoked'thought-probe trials. In block 1, these trials followed ISIs of 2, 4, 6, 8, and 10 s, while in block 2, they followed ISIs of 1, 3, 5, 7, and 9 s. These yoked trials presented participants with the blue zeros during the waiting period that were replaced by a thought probe (described below) rather than counting upward. The dependent measure was the mean RT of the slowest 20% trials (e.g., Unsworth & Robison, 2016; Welhaf & Kane, 2024b). The task took approximately 15 min to complete.

*Continuous Temporal Expectancy Task (CTET;* O’Connell et al., 2009). Participants viewed a series of stimuli onscreen, with 90% of stimuli in a block presented for a standard duration (non-targets), and target stimuli presented for an “oddball” duration; participants pressed the “5” key on the numeric keypad only for target stimuli with an oddball duration. Each stimulus was a matrix with all the internal squares shaded in half along a diagonal; within a matrix, all squares were shaded the same way (e.g., below the diagonal running from top left to bottom right), but between consecutive matrices the shading changed (e.g., above the diagonal running from bottom left to top right).

Participants completed a practice block of 40 trials with four targets, and then two experimental blocks, each presenting 200 trials (each block also began with four unanalyzed buffer trials) of which 20 were targets. In the first block, non-target stimuli appeared for a brief duration (600 ms) while target stimuli appeared for a longer duration (1200 ms). In the second block, non-targets again appeared for 600 ms, and targets appeared for an even longer duration (1500 ms). We calculated dʹ for each block and because these two scores were highly correlated (*r* =.74) we created an overall CTET dʹ score by *z*-scoring dʹ from each block and then averaging the *z*-scores together. The task took approximately 15 min to complete.

*Metronome Response Task (MRT;* Seli et al., 2013*).* Participants were instructed to tap synchronously with a steady beat of clicks presented through Koss UR-20 over-ear Headphones by pressing the spacebar in time with each click. Each trial began with 650 ms of silence, followed by a 75 ms click, and then 575 ms of silence, resulting in a total trial duration of 1300 ms. Participants completed 12 blocks of 36 trials, for a total of 432 trials with a thought probe presented randomly in each block (see below). The dependent measure was the rhythmic response time (RRT; Seli et al., 2013). RRT was calculated using a five-trial moving window (excluding the first five trials, the five trials after thought probes, and the five trials after any omission errors). The variance for each moving window was calculated and then log-transformed to account for high skew. This transformed value was then averaged within participants to create an RRT score. The task took approximately 15 min to complete.

**Fig. 1:**
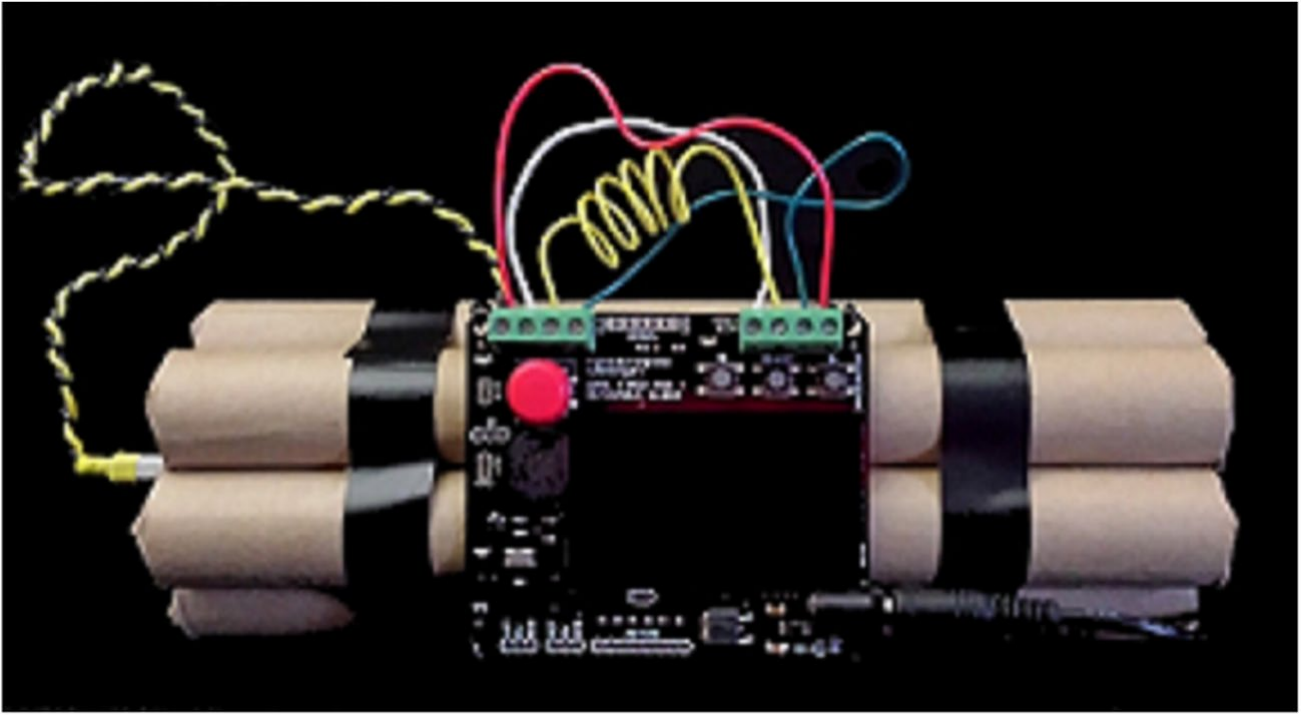
Display of Motivation PVT background. The zeros (000.00) appeared within the black square in the center of the time bomb display

*Motivation Psychomotor Vigilance Task (MotPVT).* The MotPVT was nearly identical to the PVT described above, with the same ISI distribution, but with changes introduced to increase motivation and interest. Rather than instruct participants to respond to stop a stopwatch, they were shown a picture of a time bomb device (see Fig. 1) with a row of 0 s in the center (“00.000”); the display of 0 s from the standard PVT was imposed on the blank black rectangle depicted in Fig. 1. Participants played the role of an FBI agent responsible for diffusing a series of Linked time bombs at multiple local universities; their goal was to stop the timer on each trial before it hit 350 ms, or that bomb would explode. If participants failed to respond at the 350-ms mark, the counter continued to count until a response; participants did not receive explicit feedback that they missed the target time, beyond the displayed RT upon responding. In addition to this gamification of the PVT, we also incentivized participants to perform well by informing them that if they met a certain (unspecified) performance criterion in the task, they would be able to leave the experimental session early (for a similar manipulation, see Seli et al., 2019); they were also told they would receive a chocolate bar or lollipop of their choosing for reaching the performance criterion. All participants saw a screen at the end of the task indicating that they reached the criterion and could now leave the session early, with the candy of their choice. As with the standard PVT (e.g., Unsworth & Robison, 2016; Welhaf & Kane, 2024b), the dependent measure was the mean RT of participants’ slowest 20% of trials. The task took approximately 15 min to complete.^1^

#### Self-report-based attention consistency measures

Participants responded to thought probes in four tasks: the SART (24 probes), the PVT (10 probes), the MRT (12 probes), and the MotPVT (10 probes). To increase the diversity of our mind-wandering assessment (in parallel with our selection of varied performance measures, described above), we presented two types of content-oriented thought probes across tasks.

In the PVT and MotPVT, each probe asked participants to classify their immediately preceding thoughts into one of eight thought content categories (Kane et al., 2016, 2017, 2021a, 2021b; McVay & Kane, 2009, 2012a; Welhaf et al., 2022). The response options were: (1) “The task” (thoughts about the stimuli or responses); (2) “Task experience/performance” (thoughts about one’s task performance); (3) “Everyday things” (thoughts about normal life concerns, goals, and activities); (4) “Current state of being” (thoughts about one’s physical, cognitive, or emotional state); (5) “Personal worries” (thoughts about current worries); (6) “Daydreams” (fantastical, unrealistic thoughts); (7) “External environment” (thoughts about task-unrelated things or events in the testing room); (8) “Other.” Only the phrases in quotes above appeared on the probe screens. TUT rates were operationalized in these two tasks as the proportion of probes with responses 3–8.

In the SART and MRT, the probes also asked about the content of participants’ immediately preceding thoughts, but here they classified the temporal orientation of that content (McVay & Kane, 2012b; Robison & Unsworth, 2017; Smallwood et al., 2009; Unsworth & McMillan, 2013). Specifically, the response options were: (1) “The task” (about the stimuli/response); (2) “Task experience/performance” (about one’s performance); (3) “Something from the past” (about life events in the recent or distant past); (4) “Something in the future” (about events or activities in the near or distant future); (5) “Something in the present” (about immediate states or events; such as current state of being or things/events in the testing room); (6) “Something non-temporal/outside of time*”* (e.g., about fantasy/imaginations, abstract ideas, inner music, knowledge). TUT rates were operationalized as the proportion of probes with responses 3–6.

#### Task motivation and interest

In four tasks (Avengers AX-CPT, SART, PVT, and MotPVT), participants answered a series of questions about conative factors, such as their performance motivation and task interest (e.g., *I expect the content of this task will be interesting*; *The task brought out my competitive drives*). We drew 14 items from the motivation subscale of Dundee State Stress Questionnaire (DSSQ; Matthews et al., 2002). Seven items were presented immediately following the practice trials of the task and before the critical trials, and the remaining seven items were presented immediately upon task completion. Each item was rated via mouse click on a 1–5 Likert scale, with points labeled, *Not at all, A little bit, Somewhat, Very much, Extremely* (or, participants could press a separate box to skip the item). The dependent measures were two-factor scores derived from an exploratory factor analysis (EFA) on each set of DSSQ items for each task separately (described in detail in the Results section).

## RT cleaning procedures

For all tasks with RT-based dependent measures (i.e., SART, MRT, and both PVTs), we examined individual participants’ RT distributions for outliers, following identical procedures to our previous work (e.g., Rodriguez-Boerwinkle et al., 2024; Welhaf et al., 2020; Welhaf & Kane, 2024b). Specifically, for each participant and each task, we first removed RTs for error, post-error, and post-thought-probe trials. Next, we removed RTs that were likely anticipations (< 200 ms). Finally, from the remaining trials, we calculated a participant-specific outlier cutoff equal to their Median RT + 3*IQR. RTs larger than this value were replaced with this value. The median number of RTs this affected for each task was 3 (range = 0–26) in the SART, 2 (range = 0–41) in the MRT, 2 (range = 0–8) in the PVT, and 1 (range = 0–7) in the MotPVT.^2^

## Data exclusions

As in our prior work (Kane et al., 2016; Welhaf & Kane, 2024b), we excluded data from participants who exhibited behaviors during the session that might suggest a failure to engage with the tasks as instructed, based on experimenter session notes, while blinded to participants’ performance data and thought probe responses (research assistants were instructed to monitor and note such behaviors). Data from 12 participants were excluded case-wise for falling asleep in multiple tasks; data from two additional participants were excluded for indicating they were not native English speakers (and so may have had difficulty understanding task instructions).

We also implemented several task-wise exclusions. For the AX-CPT, many participants (approximately 50% of the sample) had near floor overall cue accuracy, suggesting they did not understand the task instructions to respond to both the cue and probe on each trial. We therefore dropped all AX-CPT data for participants with overall cue accuracy below 40% (*n* = 175 excluded). This decision was made during data processing and was based on examining the distribution of cue accuracy scores. For the SART, we excluded participants with go-trial accuracy rates < 70%, suggesting failure to understand or follow task instructions (*n* = 29; for similar approaches see Welhaf & Kane, 2024a, 2024b; Unsworth et al., 2021). For the PVT and MotPVT, we excluded data from participants who had ten or more RTs removed that were either < 200 ms or outside their Median RT + 3*IQR, or from participants whose *M* RT of their slowest 20% of trials was > 1000 ms (i.e., for each participant, all RTs were sorted from shorted to longest and binned into five quantiles, and *M* RT was calculated for each quantile bin; see Robison et al., 2023; Robison & Nguyen, 2023 for a similar approach); we therefore excluded PVT data for 23 participants and MotPVT data for five participants.

For the MRT, we excluded data from 24 participants with an omission rate > 10%, consistent with previous work (see Seli et al., 2013). Finally, we excluded data from 27 participants in the CTET who exhibited an exceedingly high false alarm rate in either block (> Median + 3*IQR), suggesting they responded to most stimuli rather than just the longer-than-normal target stimuli; the average false alarm rate from excluded participants was ⁓14, whereas *M* false alarm rates in other CTET studies are often below 3% (e.g., Brosnan et al., 2018; Gray et al., 2015; Pinggal et al., 2022). Following these task-wise exclusions, we removed all data from ten participants who had missing data on at least four of the seven analyzed tasks.

Following Welhaf and Kane (2024b), we implemented a three-step procedure to ensure univariate and multivariate normality. First, we censored outlying scores for each measure. Outliers were defined as cases outside the sample Median ± 3*IQR and were censored to the Median ± 3*IQR value for that measure. Next, we standardized scores on each measure to the sample and replaced values > 4 SDs away from the sample mean with a value equal to 3.99 SDs. This procedure affected only a single case, on CTET Block 1 dʹ. Finally, consistent with our previous work (e.g., Rodriguez-Boerwinkle et al., 2024; Welhaf & Kane, 2024b), we screened the remaining sample for multivariate outliers. We used the *Routliers* package (Leys et al., 2013) to identify participants with a Mahalanobis distance greater than 12, which corresponds to points that are roughly in the 1% of most extreme observations, resulting in the removal of an additional seven participants’ data.

## Modeling approach and fit statistics

Our primary analytic approach relied on confirmatory factor analyses (CFA) and structural equation models (SEM). Given that our sample had missing data, we used full information maximum likelihood for model estimation. For all CFAs and SEMs, we report several fit statistics: χ^2^ (and associated degrees of freedom and *p* value of the model); χ^2^/*df*, where values < 3 indicate adequate fit; Comparative Fit Index (CFI) and Tucker–Lewis Index (TLI), with values >.90 suggesting adequate fit; root mean square error of approximation (RMSEA) and its associated 90% confidence interval [90% CI], with values <.10 indicating adequate fit; standardized root mean square residual (SRMR), with values <.08 indicating adequate fit (Hu & Bentler, 1999). All latent variable models were run in R using the *lavaan* package (Rosseel, 2012). We conducted EFA using the *psych* package (Revelle, 2017) to analyze the underlying factor structure of each Motivation DSSQ. We saved factor scores and used them as observed conative variables in subsequent CFA and SEMs. These factor scores were saved using the “tenBerge” method in the factor.analysis function (Grice, 2001; Ten Berge et al., 1999) as this method is shown to maintain the correlation between factors).

## Transparency and openness

Data and an R Markdown file to reproduce all reported analyses can be found on the Open Science Framework (https://osf.io/jqtgb/). This exploratory study was not preregistered. We interpret zero-order correlations using rules of thumb based on prototypical effect sizes in psychological individual differences studies where *r* =.10,.20, and.30 represent relatively small, medium, and large effect sizes, respectively (Gignac & Szodorai, 2016).

## Results

The final sample of 402 undergraduates (*M*_*age*_ = 19.17 years, *SD*_*age*_ = 1.89; range: 18–32) primarily self-identified as female (68% female; 25% male, 6% non-binary, *n* = 3 missing). The racial breakdown of the sample was characteristic of similar studies using student samples at our two sites: 36% White, 34% Black or African American, 12% Hispanic or Latino/a, 10% multiracial, 3% South Asian, 2% East Asian, 2% Middle-Eastern, Arab, or North African, and < 1% Native Hawaiian or Pacific Islander.

Descriptive statistics (and *n*s) for each measure are presented in Table 1. All variables exhibited acceptable levels of skewness (<|3|) and kurtosis (<|10|; Brown, 2006) for the purpose of latent variable analyses. Zero-order correlations and reliabilities (all coefficient α >.70; *Mdn* α =.88) for the performance and self-report indicators are presented in Table 2. As expected, performance-based attention consistency measures tended to correlate with one another (median |*r*|=.16; *r*_range_ =.10–54), as did the self-report attention consistency measures (i.e., TUT rates; median *r* =.34; *r*_range_ =.31–45). Thus, our task battery appeared to yield stable and reliable individual differences in performance and thought-report indicators of attention consistency.

**Table 2:** Correlation matrix of attention consistency measures and DSSQ conative factor scores

	1	2	3	4	5	6	7	8	9	10
1. SART RTsd	*.91*									
2. PVT Slowest 20%	.37***	*.90*								
3. MRT RRT	.35***	.37***	*.96*							
4. MotPVT Slowest 20%	.35***	.64***	.31***	*.87*						
5. AX-CPT dʹ	–.32***	–.22**	–.15*	–.17**	*.91*					
6. DVG dʹ	–.32***	–.28***	–.30***	–.27***	.26***	*.93*				
7. SART Omissions	.10^	.12*	.16**	.10*	–.29***	–.32***	*.89*			
8. CTET dʹ Composite	–.36***	–.42***	–.38***	–.32***	.22***	.40***	–.21***	*.83*		
9. SART TUT	.15**	.16**	.09	.07	–.01	–.11*	.16**	–.21***	*.82*	
10. PVT TUT	.13*	.37***	.14**	.25***	–.14*	–.16**	.11*	–.14**	.45***	*.69*
11. MRT TUT	–.04	.03	–.08	.02	.16*	.14**	–.04	.01	.34***	.33***
12. MotPVT TUT	.14**	.17**	.14**	.30***	.02	.02	.07	.00	.31***	.43***
13. AX-CPT Factor 1	–.10	–.27***	–.08	–.24***	.09	.12^	.03	.13*	–.17*	–.21**
14. AX-CPT Factor 2	–.06	–.05	–.06	–.06	–.03	–.02	.04	.10	–.20**	–.07
15. SART Factor 1	–.16**	–.18***	–.03	–.22***	.00	.08	–.06	.10^	–.26***	–.17**
16. SART Factor 2	–.02	–.09^	.01	–.08	–.06	–.07	–.10*	.08	–.28***	–.16**
17. PVT Factor 1	–.12*	–.29***	–.10^	–.26***	.01	.09^	–.04	.14**	–.18***	–.27***
18. PVT Factor 2	–.10^	–.23***	–.06	–.14**	.00	.00	–.06	.20***	–.18***	–.21***
19. MotPVT Factor 1	–.16**	–.25***	–.11*	–.39***	.01	.07	–.01	.15**	–.12*	–.17***
20. MotPVT Factor 2	–.08	–.16**	–.10^	–.23***	–.02	–.01	–.04	.18***	–.09^	–.13*

	11	12	13	14	15	16	17	18	19	
1. SART RTsd										
2. PVT Slowest 20%										
3. MRT RRT										
4. MotPVT Slowest 20%										
5. AX-CPT dʹ										
6. DVG dʹ										
7. SART Omissions										
8. CTET dʹ Composite										
9. SART TUT										
10. PVT TUT										
11. MRT TUT	*.83*									
12. MotPVT TUT	.34***	*.75*								
13. AX-CPT Factor 1	–.03	–.10	–							
14. AX-CPT Factor 2	–.18**	–.08	.28***	–						
15. SART Factor 1	–.05	–.12*	.76***	.30***	–					
16. SART Factor 2	–.16**	–.16**	.19**	.72***	.44***	–				
17. PVT Factor 1	–.02	–.12*	.70***	.27***	.79***	.37***	–			
18. PVT Factor 2	–.09^	–.15**	.23***	.63***	.34***	.72***	.50***	–		
19. MotPVT Factor 1	–.03	–.27***	.70***	.31***	.72***	.30***	.76***	.37***	–	
20. MotPVT Factor 2	–.12*	–.29***	.19**	.55***	.20***	.56***	.29***	.68***	.46***	

^ *p* <.10, * *p* <.05, ** *p* <.01, *** *p* <.001. Split-half reliabilities based on odd–even trials are presented on the diagonal. SART = Sustained Attention to Response Task; PVT = Psychomotor Vigilance Task; MRT = Metronome Response Task; MotPVT = Motivation PVT; DVG = Degraded Vigilance Task; CTET = Continuous Temporal Expectancy Task; RTsd = Intra-individual RT standard deviation; Slowest 20% = Mean RT of Slowest 20% of Trials; RRT = Rhythmic Response Time; TUT = proportion of Task-Unrelated Thoughts in task.

## Testing the factor structure of attention consistency measures

Our main goal for the current battery of attention consistency measures was to provide a broader construct-valid assessment of both performance-based and self-report-based attention consistency factors than in prior studies. We first fit a 4-factor model with each factor representing a different (but correlated) measurement approach, that is, factors for performance accuracy, performance RT variability, TUT rates from content-category probes, and TUT rates from temporal-content probes. We allowed residual correlations among the following pairs of indicators based on our previous work (Welhaf & Kane, 2024b) and modification indices: SART RTsd and Omissions, the *M* RT of the slowest 20% of trials in both the standard and Motivation PVT, TUT rates from each of the PVTs with their respective performance indicator. Although this model adequately fit the data (see Table 3 for all model fit statistics),^3^ estimation of the model produced a warning that the latent variable covariance matrix was not positive definite. Inspection of the model indicated that the two performance factors correlated extremely strongly, at *r* = – 90 [95% CI = –1.00, – 80]: Less accurate participants were also more variable in their RTs. Likewise, the two thought-report factors were correlated at *r* =.95 [95% CI =.80, 1.09]: Participants who reported more TUTs to content-based thought probes also reported more TUTs to temporal-orientation-based thought probes. Given these near-perfect correlations, we could not further parse these two broad forms (performance and self-report) of attention consistency measurement; all performance measures were then modeled as a single factor, and all TUT rates were modeled as a single factor.^4^

**Table 3:** Fit statistics for latent variable models

Model	χ^2^ (*df*)	*p* value	χ^2^/*df*	CFI	TLI	RMSEA [90% CI]	SRMR
**Measurement models**							
4-Factor*	118.571 (46)	<.001	2.58	.929	.898	.063 [.049,.077]	.062
3-Factor (Combined Performance)*	130.670 (49)	<.001	2.67	.920	.892	.064 [.051,.078]	.065
3-Factor (Combined Self-Report)*	125.330 (47)	<.001	2.67	.923	.892	.064 [.051,.078]	.064
2-Factor	131.843 (49)	<.001	2.69	.919	.891	.064 [.052,.078]	.066
1-Factor	335.054 (50)	<.001	6.70	.720	.631	.119 [.107,.131]	.101
Bifactor	75.628 (38)	<.001	1.99	.963	.936	.050 [.033,.066]	.040
Hierarchical	131.843 (49)	<.001	2.69	.919	.891	.064 [.052,.078]	.066
Refined 2-Factor	51.170 (23)	.001	2.15	.966	.947	.055 [.035,.076]	.037
Refined Bifactor	–		–	–	–	–	–
Refined Hierarchical	51.170 (23)	.001	2.15	.966	.947	.055 [.035,.076]	.037
**Confirmatory factor analyses**							
2-Factor	455.056 (156)	<.001	2.92	.906	.885	.069 [.062,.076]	.069
Hierarchical	457.041 (158)	<.001	2.89	.906	.887	.069 [.061,.076]	.070
Refined 2-Factor	341.606 (106)	<.001	3.22	.921	.899	.074 [.066,.083]	.059
Refined Hierarchical	342.958 (108)	<.001	3.18	.921	.901	.074 [.065,.082]	.061

* Model estimation produced a warning that the latent variable covariance matrix was not positive definite

**Fig. 2:**
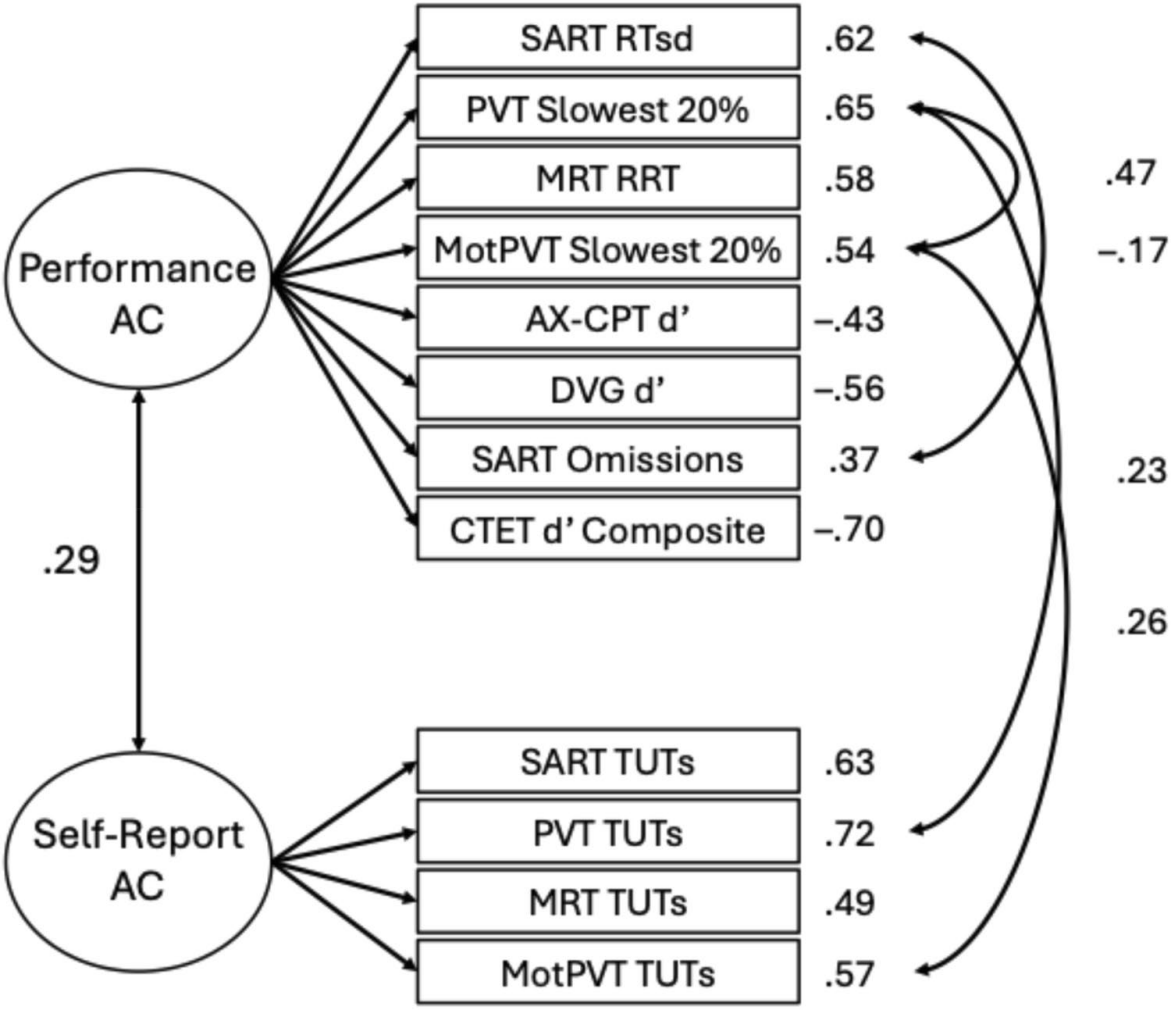
Two-factor measurement model of attention consistency, with one factor reflecting performance-based measures (“Performance AC”) and one factor reflecting self-report-based measures (“Self-Report AC”). Factor loadings are presented next to each observed indicator. Solid lines represent significant paths at *p* <.05

**Fig. 3:**
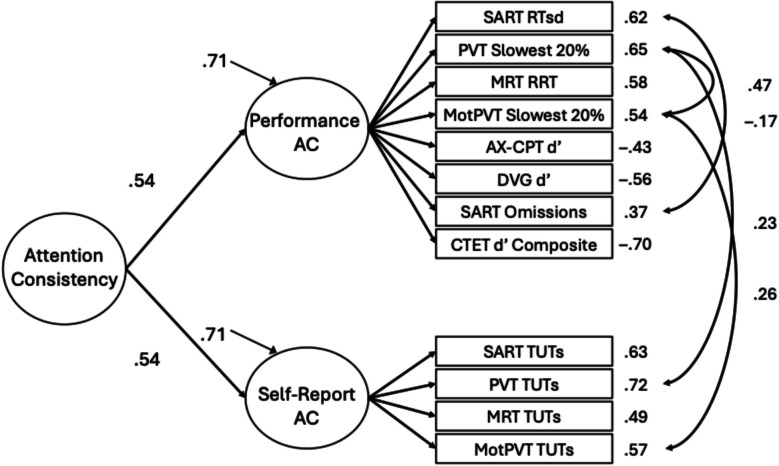
Measurement model for hierarchical attention consistency (AC) model. Factor loadings are displayed next to indicator measures. Solid lines represent significant paths at *p* <.05. Residual correlations align with the peak of the connecting arrows

Next, we fit a two-factor model consistent with Welhaf and Kane (2024b), with separate factors for performance-based and self-report measures. This model adequately fit the data, with no warnings (see Table 3). Individual differences in performance measures and self-report measures of attention consistency were positively correlated (*r* =.29 [95% CI:.16,.42], *p* <.001), replicating the association previously reported (see Fig. 2).^5^ As in Welhaf and Kane (2024b), this moderate correlation confirmed that these two indicator types of attention consistency are not redundant. Instead, they may reflect different degrees or aspects of attentional disengagement, with each having their own (considerable) sources of measurement error Fig. 3.

Having re-established this moderate correlation with a new task battery, we next attempted to model a higher-order, general factor of sustained attention consistency (Welhaf & Kane, 2024b). We approached this in two ways, first testing a bifactor model where the shared variance among all indicators was explained by a “common” attention consistency factor and all performance and thought-report indicators also loaded onto respective (and orthogonal) “residual” factors. These residual factors reflected variance unique to the performance or thought-report indicators of attention consistency after accounting for their shared variance in the common factor. This bifactor model appeared to fit the data (see Table 3 for fit statistics), but upon inspection of factor loadings, none were significant for the performance-residual factor (although the TUT-rate-residual factor appeared coherent). Thus, the performance-based indicators saturated the general factor, consistent with some bifactor models reported in Welhaf and Kane (2024b). Because of this, we focus instead on the hierarchical model in which a second-order attention consistency latent variable was modeled as the shared variance between the first-order performance and self-report latent variables. As in Welhaf and Kane (2024b), we set the unstandardized paths to be equal because the higher-order factor had only two indicators. This model adequately fit the data (see Table 4 for factor loadings for all models; see Fig. 3 for the resulting model).

The first-order measurement-type factors both loaded significantly onto the second-order attention consistency factor (performance β =.54, self-report β =.54). Note, however, that the residual variances for the first-order factors were large (performance ζ =.71, self-report ζ =.71). Despite fitting the data well, there was a large portion of unexplained variance in the measurement-type factors, as is expected from the two-factor model showing only a moderate correlation between the performance and self-report factors. We retained this hierarchical measurement model for subsequent tests of the association between attention consistency and different facets of motivation Tables 4 and 5.

**Table 4:** Factor loadings of latent variable models

Construct and measures	Model					
	Two-factor Measurement	Hierarchical Measurement	Two-factor CFA	Hierarchical CFA	Two-factor SEM	Hierarchical SEM
**Performance AC**						
SART RTSD	.62 (.04)	.62 (.04)	.62 (.04)	.63 (.04)	.63 (.04)	.63 (.04)
PVT Slowest 20%	.65 (.04)	.65 (.04)	.66 (.04)	.67 (.04)	.66 (.04)	.67 (.04)
MRT RRT	.58 (.04)	.58 (.04)	.58 (.04)	.58 (.04)	.55 (.04)	.56 (.04)
MOT PVT Slowest 20%	.54 (.05)	.54 (.05)	.55 (.05)	.56 (.05)	.58 (.05)	.58 (.05)
AX-CPT d’	–.43 (.07)	–.43 (.07)	–.41 (.07)	–.41 (.07)	–.41 (.07)	–.41 (.07)
DVG d’	–.56 (.04)	–.56 (.04)	–.55 (.04)	–.55 (.04)	–.55 (.04)	–.55 (.04)
SART Omissions	.37 (.05)	.37 (.05)	.36 (.05)	.36 (.05)	.36 (.05)	.36 (.05)
CTET d’	–.70 (.03)	–.70 (.03)	–.70 (.03)	–.71 (.03)	–.70 (.03)	–.71 (.03)
**Self-Report AC**						
SART TUTs	.63 (.05)	.63 (.05)	.64 (.05)	.63 (.05)	.64 (.05)	.63 (.05)
PVT TUTS	.72 (.05)	.72 (.05)	.72 (.05)	.72 (.05)	.72 (.05)	.72 (.05)
MRT TUTS	.49 (.05)	.49 (.05)	.48 (.05)	.48 (.05)	.48 (.05)	.48 (.05)
MOT PVT TUTS	.57 (.06)	.57 (.06)	.58 (.05)	.57 (.05)	.58 (.05)	.57 (.05)
**Motivation Factor 1**						
AX-CPT Factor 1			.82 (.03)	.82 (.03)	.82 (.03)	.82 (.03)
SART Factor 1			.89 (.02)	.89 (.02)	.89 (.02)	.89 (.02)
PVT Factor 1			.88 (.02)	.88 (.02)	.88 (.02)	.88 (.02)
MOT PVT Factor 1			.84 (.02)	.84 (.02)	.84 (.02)	.84 (.02)
**Motivation Factor 2**						
AX-CPT Factor 2			.76 (.03)	.76 (.03)	.76 (.03)	.76 (.03)
SART Factor 2			.82 (.02)	.81 (.02)	.82 (.02)	.81 (.02)
PVT Factor 2			.88 (.02)	.88 (.02)	.88 (.02)	.89 (.02)
MOT PVT Factor 2			.73 (.03)	.73 (.03)	.73 (.03)	.73 (.03)

AC = Attention Consistency; SART = Sustained Attention to Response Task; PVT = Psychomotor Vigilance Task; Mot = Motivation; MRT = Metronome Response Task; RRT = Rhythmic Response Time; DVG = Degraded Vigilance Task; CTET = Continuous Temporal Expectancy Task; TUT = Rate of Task-Unrelated Thoughts.

**Table 5:** Factor loadings from exploratory factor analysis on dssq conative scale items for each task

Item	AX-CPT			SART			PVT			MotPVT		
	Factor 1	Factor 2	*h*^2^	Factor 1	Factor 2	*h*^2^	Factor 1	Factor 2	*h*^2^	Factor 1	Factor 2	*h*^2^
Q1		.48	.30		.46	.36	.36	.39	.42	.45	.38	.50
Q2	.40		.15	.49		.20	.48		.18	.61		.30
Q3		.50	.24		.70	.40		.70	.38		.79	.50
Q4	.54		.32	.77		.55	.76		.59	.79		.61
Q5		.68	.45		.75	.55		.84	.62		.80	.59
Q6	.65		.45	.78		.57	.76		.63	.79		.65
Q7	.46		.36	.58		.47	.56		.55	.68		.60
Q8		.65	.42		.75	.53		.67	.50		.64	.49
Q9	.83	–.33	.64	.82		.63	.88		.66	.85		.62
Q10	.84		.69	.80		.70	.87		.74	.85		.74
Q11	.37	.35	.33		.56	.39	.33	.39	.39	.47	.33	.47
Q12	.54		.31	.38		.29	.41		.26	.36		.23
Q13	.68		.44	.63		.45	.74		.50	.73		.53
Q14	.70		.49	.69		.53	.75		.61	.83		.66

Only factor loadings ≥.30 are displayed. *h*^2^ = communality score.

## Exploratory factor analysis of DSSQ conative items

We first conducted a series of EFA models on each set of DSSQ Motivation scale items, for each task, given that the original scale was split into pre- and post-task items. Before examining factor structures, we examined item-level correlations within each task. Item correlations were moderate to strong, but they appeared to group themselves into two clusters (item-level correlations for each task can be found in Supplemental Material). To confirm these patterns, we submitted each set of items to EFA with promax (oblique) rotation. Examination of scree plots and eigenvalues > 1.0 suggested a two-factor solution was appropriate for each task. The resulting factor loadings for each item, for each task, and their communality estimate are provided in Table 5.

The resulting two-factor solution generally maps on to two of the primary subscales of the DSSQ Motivation scale: success motivation (Factor 1; e.g., “*I am motivated to do this task*”, “*I put a lot of effort into my performance*”) and task interest (Factor 2; e.g., “*I expect the content of the task will be interesting*”, “*I would have rather spent my time doing something else other than this task”*). Within-task factor correlations (see Table 2) suggested that the two resulting factors were moderately-to-strongly correlated with each other, but not redundant (Mdn *r* =.46); the correlations across tasks for Factor 1 (success motivation; Mdn *r* =.73) and Factor 2 (task interest; Mdn *r* =.65) tended to be stronger than the between-factor correlations within tasks.

## Testing the associations between conative factors and attention consistency

**Fig. 4:**
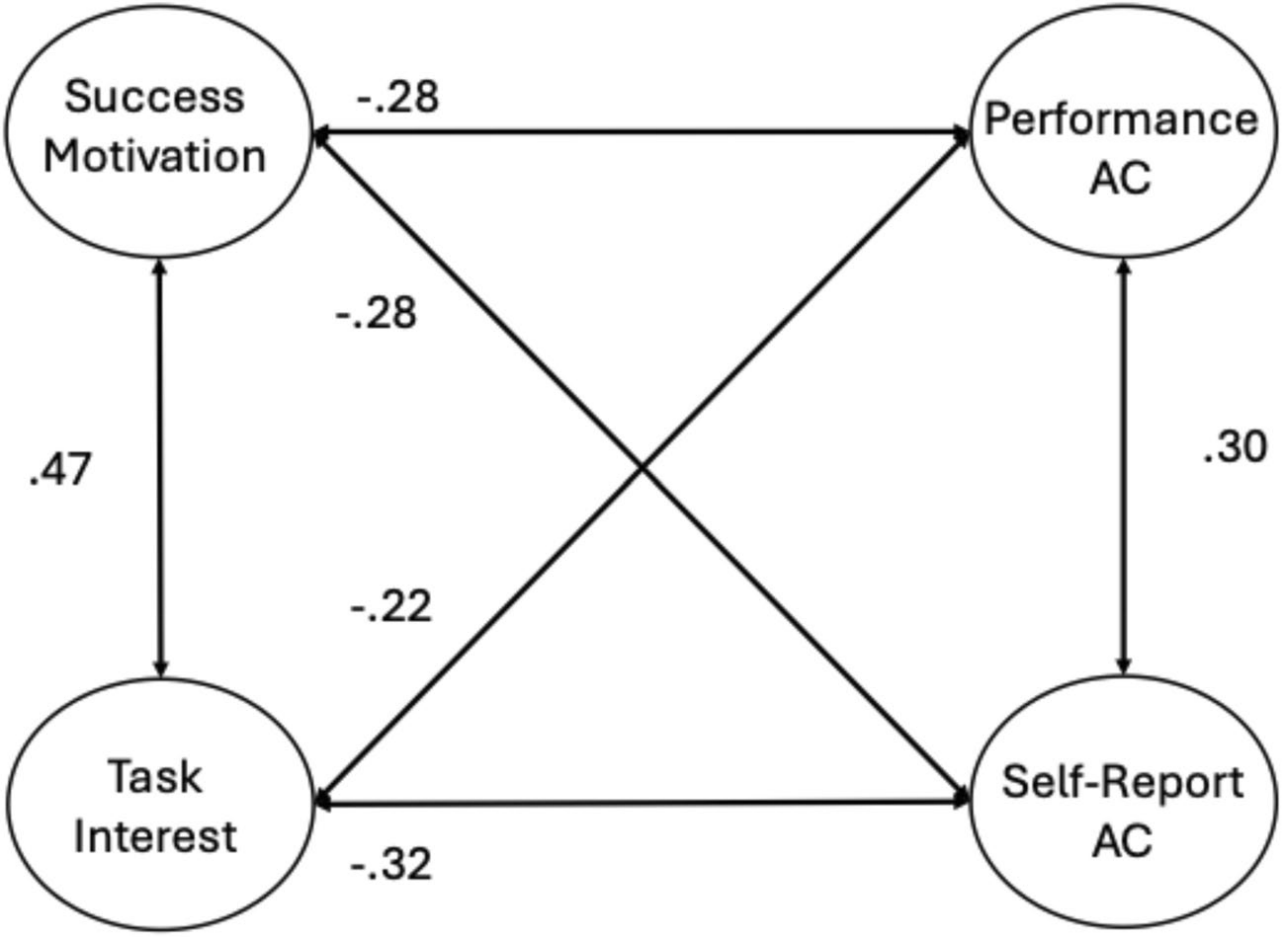
CFA of two-factor attention consistency (AC) model (with correlated performance and self-report factors) with the two DSSQ Motivation factors. Standardized path estimates are presented. For clarity, factor loadings are presented in Table 4. Solid lines represent significant paths at *p* <.05

To examine the relations between attention consistency and the two underlying conative factors, we ran a series of CFA and SEM analyses. Our first CFA examined the associations between the DSSQ conative factors and the measurement-specific attention consistency factors from the two-factor sustained attention model. The two DSSQ factors were loaded by the saved factor scores for each task, from the EFA analyses above, as the manifest variables (residual correlations between the success motivation and task interest measures from the same task were included and ranged from *r* =.22–55; see Appendix Fig. 9). Figure 4 presents the resulting model (model fit statistics can be found in Table 3 and factor loadings in Table 4). As expected, the two conative factors were positively correlated with each other (*r* =.47), and each conative factor correlated moderately with each of the attention consistency factors. Participants with higher success motivation scores and higher task interest scores showed fewer or weaker performance failures and reported fewer TUTs within the probed tasks.

**Fig. 5:**
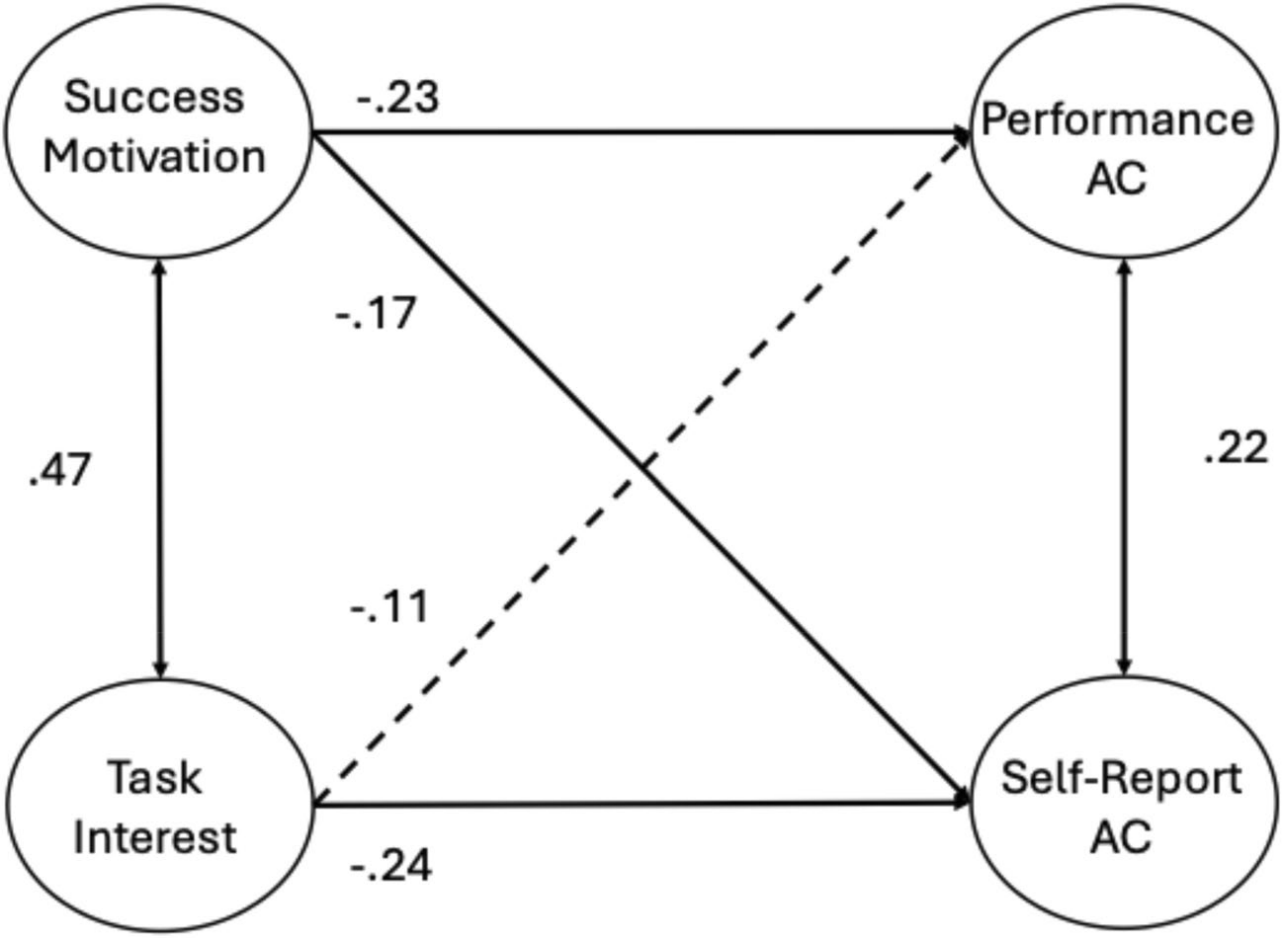
SEM of two-factor attention consistency model (AC) with the two DSSQ Motivation factors. Standardized path estimates are presented. For clarity, factor loadings are presented in Table 4. Solid lines represent significant paths at *p* <.05. Dashed lines represent non-significant paths

We then used SEM to assess the unique relations between the DSSQ conative and measurement-specific attention consistency factors. The resulting model is displayed in Fig. 5 (factor loadings are presented in Table 4). Success motivation uniquely predicted both aspects of attention consistency and did so to a similar degree. Task interest, in contrast, only significantly predicted unique variance in the self-report attention consistency factor but not in the performance factor (*ß* = −.11, *p* =.094).

**Fig. 6:**
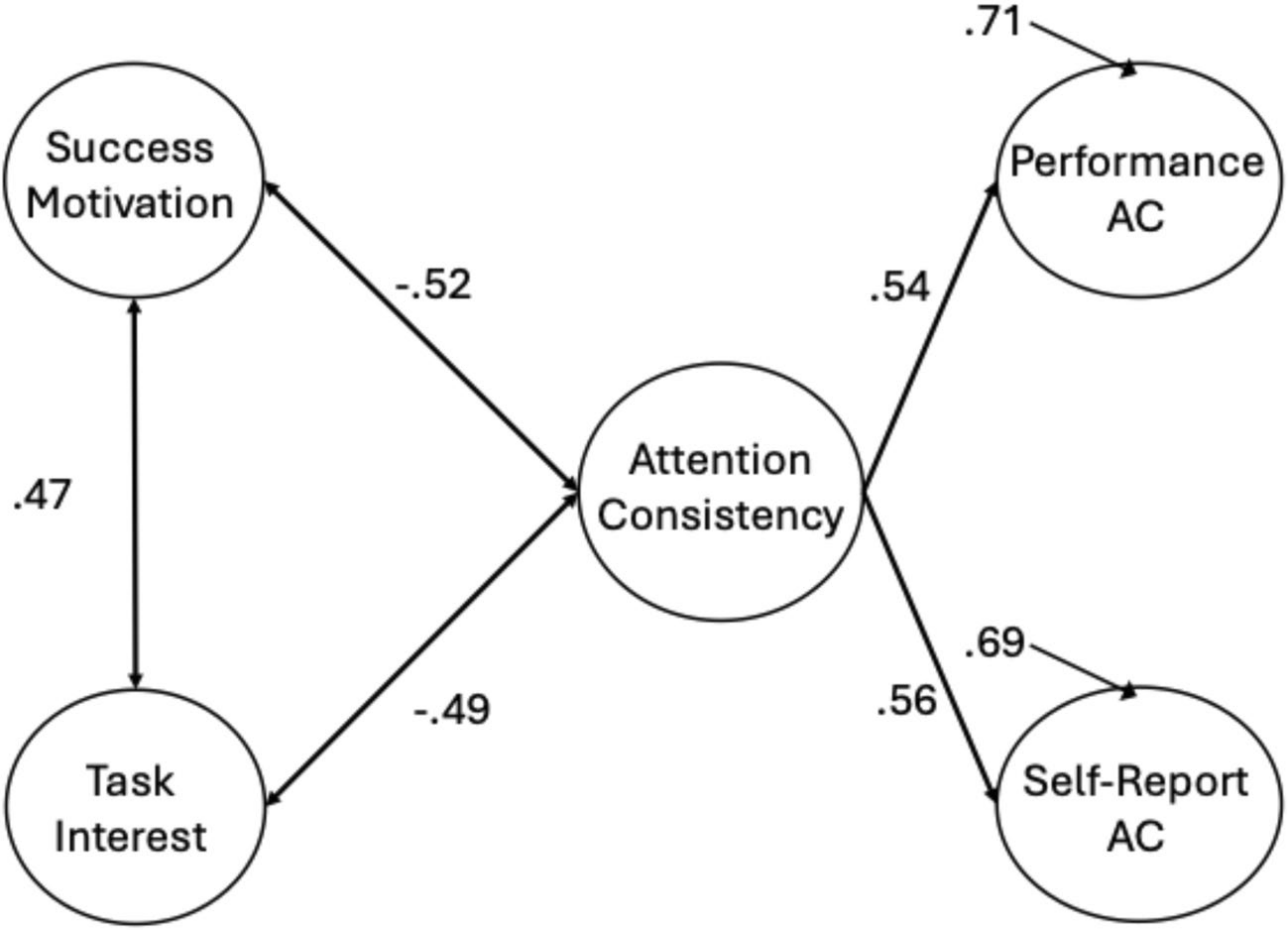
CFA of the hierarchical attention consistency (AC) model with the two DSSQ Motivation factors. Standardized path estimates are presented. For clarity, factor loadings are presented in Table 4. Solid lines represent significant paths at *p* <.05

Following this two-factor attention consistency model, we conducted a CFA to examine the associations between the two conative factors and the general, higher-order attention consistency factor. The resulting model is displayed in Fig. 6 (factor loadings are presented in Table 4). Again, both conative factors were significantly correlated with the higher-order attention consistency factor. Consistent with our previous work (Welhaf & Kane, 2024b), these factors were even more strongly correlated with the higher-order attention consistency factor than they were with either of the individual (method-specific) first-order factors in the previous model (≈.50 vs. ≈.25).

**Fig. 7:**
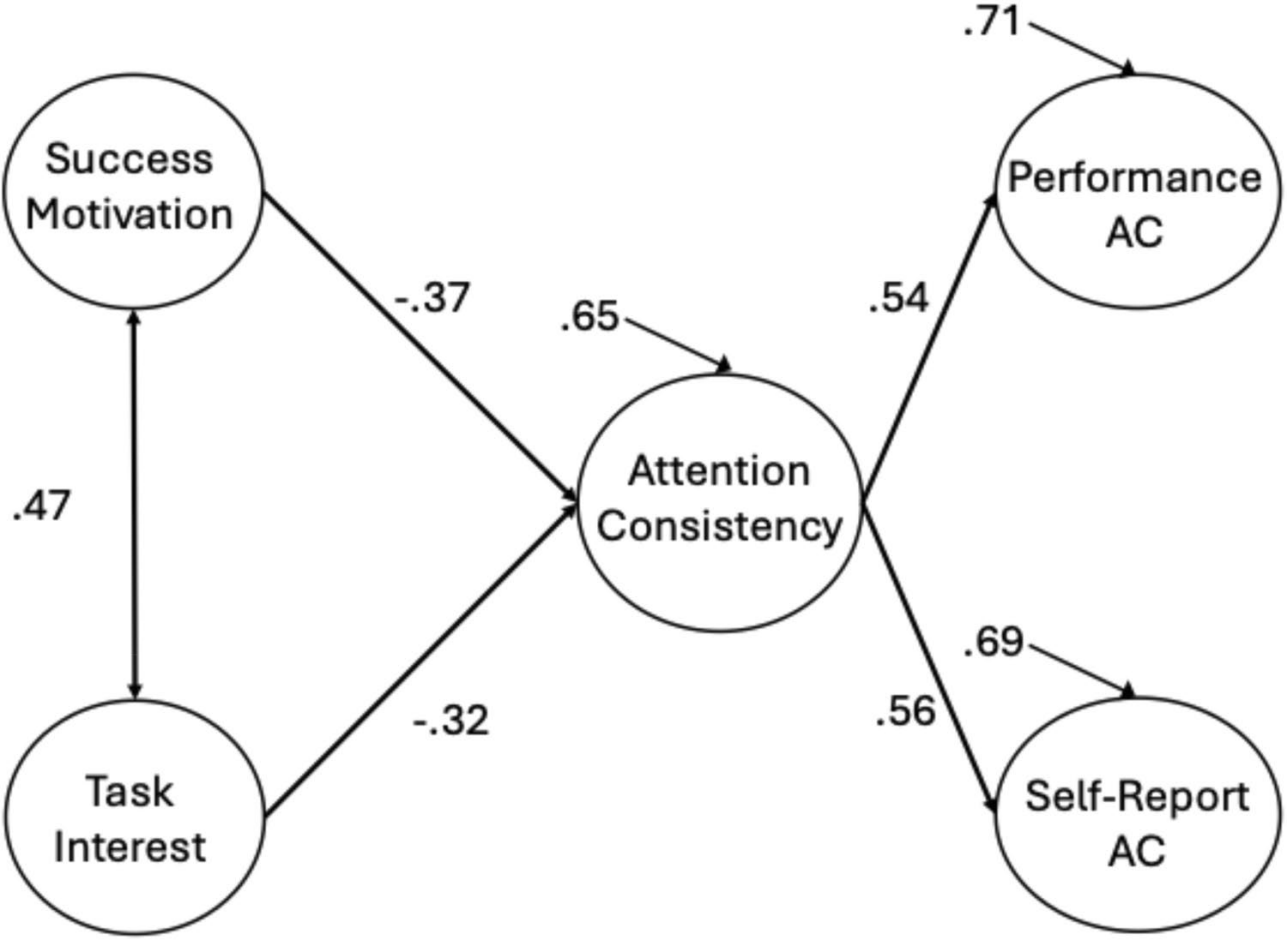
SEM of the hierarchical attention consistency (AC) model with the two DSSQ Motivation factors. Standardized path estimates are presented. For clarity, factor loadings are presented in Table 4. Solid lines represent significant paths at *p *<.05

Given the relatively strong relationships between the two conative factors with the general attention consistency factor, we next tested for any unique associations. The results of the SEM are displayed in Fig. 7 (factor loadings in Table 4). Both success motivation and task interest accounted for unique variance in general attention consistency. In contrast to the SEM with the two-factor attention consistency model, where task interest did not uniquely predict the performance-measure factor (and where the strongest significant path = −.25), it does appear to have a unique relationship with general attention consistency ability, providing further supportive evidence that the shared variance among performance and self-report attention consistency indicators is a more construct valid approach to assessing individual differences in attention consistency than is using either measurement approach on its own (note also that the strongest significant conative–attention path in the two-factor SEM = −.24, whereas the *weakest* path in the hierarchical-model SEM = −.32).

## Refining the task battery

This study’s primary objective was to use the full battery of performance and self-report measures to assess the construct of attention consistency by using a variety of stimuli, task demands, and dependent variables. Examining the zero-order correlations, however, indicated that some measures from some tasks were only weakly correlated with other measures of their intended construct. As a secondary analysis, we explored how focusing on measures that appeared to be more closely associated would improve the construct validity of our attention consistency models.

Specifically, as seen in Table 2, dʹ from the AX-CPT and SART Omissions were only weakly correlated with other performance measures, and they had several weak correlations with TUT reports (one of which was in the wrong direction). Likewise, TUT rates from the MRT did not correlate with any of the performance indicators (including from the MRT) and in multiple cases correlated in the wrong direction. Thus, we removed these three variables from the model and again tested the two-factor and hierarchical measurement models of attention consistency. Then, we examined the associations between these fine-tuned models and DSSQ conative factors.

**Fig. 8:**
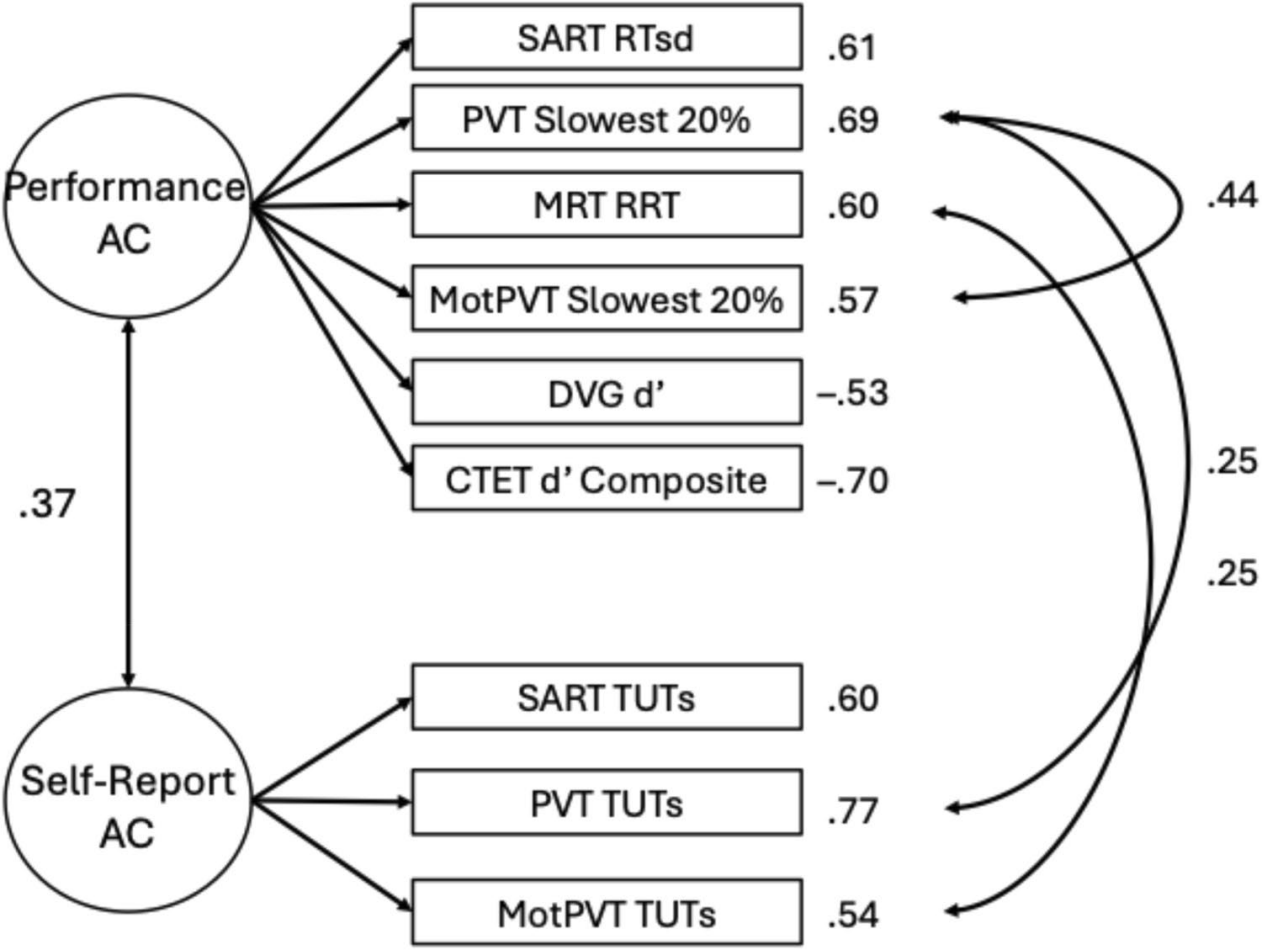
Refined two-factor model of attention consistency (AC) from the post hoc reduced task battery that eliminated apparently poor measures. Solid lines represent significant paths at *p* <.05

Fit statistics for these post-hoc refined models are reported in Table 3, which again all had adequate model fit (factor loadings are presented in Appendix Table 7).^6^ The two-factor model is presented in Fig. 8. The correlation between the performance and TUT-rate latent factors increased to *r* =.37 (compared to.29 in the original full model). Thus, the post hoc removal of poor-performing indicators strengthened the association between the factors.

Table 6 provides a head-to-head comparison of the paths from the full and refined models. The associations with the conative factors were quite like those of the full model. Specifically, both success motivation and task interest were negatively associated with the performance and self-report factors. The increases in path magnitude were generally small (range =.00–04).

**Table 6:** Path comparisons from full model to the refined models

Model	Path	Full model	Refined model	Difference
Measurement Model	Performance – Self-Report	.29	.37	.08
Two-factor CFA	Success Motivation – Performance	– .28	– .31	.03
	Success Motivation – Self-Report	– .28	– .32	.04
	Task Interest – Performance	– .22	– .24	.02
	Task Interest – Self-Report	– .32	– .32	.00
Two-factor SEM	Success Motivation – Performance	– .23	– .26	.03
	Success Motivation – Self-Report	– .17	– .21	.04
	Task Interest – Performance	– .11	– .12	.01
	Task Interest – Self-Report	– .24	– .22	– .02
Hierarchical CFA	Success Motivation – General AC	– .52	– .51	– .01
	Task Interest – General AC	– .49	– .46	– .03
Hierarchical SEM	Success Motivation – General AC	– .37	– .38	.01
	Task Interest – General AC	– .32	– .28	– .04

AC = Attention Consistency.

Turning next to the hierarchical model, once again, both lower-order factors significantly loaded onto the higher-order general attention consistency factor (performance =.60, self-report =.61). These loadings were numerically stronger than those presented in the full-battery model (performance =.54, self-report =.54), suggesting that the removed indicators contributed to measurement error. However, associations between the higher-order factor and conative factors were largely the same as in the full-battery model (see Table 6). Specifically, both success motivation and task interest were strongly correlated with the higher-order factor *r* = – 51 and – 46, respectively (which are Like those in the full model, – 52 and – 49). Finally, the SEM on the hierarchical model again indicated that both conative factors predicted unique variance in the higher-order attention consistency factor (– 38 and – 28, for success motivation and task interest, respectively, versus – 37 and – 32 in the full-battery model).

Thus, streamlining the measurement battery produced similar or better results in capturing the attention consistency construct, and it did not appear to hurt the correlations between attention consistency and conative constructs. Because we made these changes to the battery post hoc, it will be important to replicate the results from this refined battery in a new dataset. Future work will also be required to assess attention consistency correlations with a broader range of other constructs, particularly those based on task performance rather than self-report (e.g., working memory capacity, attention restraint/inhibition, fluid intelligence).

## Discussion

Individual differences in sustained attention consistency ability are assessed through two main approaches, performance-based measures and momentary self-reports of TUT experiences. The current study aimed to extend our previous work, which re-analyzed existing datasets (Welhaf & Kane, 2024b), to create a construct-valid battery of attention consistency measures from a diversity of commonly used tasks and measures. Further, we aimed to answer an unresolved construct-validation question, regarding the relationship between attention consistency and self-reported conative factors. The current study provides further evidence that attention consistency can be measured as the individual-differences covariation between performance and thought-report measures. This ability to sustain attention from moment to moment is uniquely related to both tested conative factors, success motivation, and task interest.

## Improving the breadth of attention consistency measurement

The first goal of the current study was to build a broad and construct-valid battery of sustained attention consistency measures. Our previous work (e.g., Welhaf & Kane, 2024b) relied on previously published data from two large sample individual differences studies (Kane et al., 2016; Unsworth et al., 2021), only one of which was designed to study sustained attention, and so we could only use the existing tasks and measures. Most of the performance measures from these tasks were RT-based (especially in Kane et al., 2016), so measurement error related to variation in processing speed, or speed–accuracy tradeoffs, may have diluted measurement of attention consistency.

The current battery featured diversity in stimulus and response characteristics and obtained dependent measures. Whereas both the DVG and CTET tasks, for example, presented mundane and repetitive stimuli, they both also tapped aspects of visual discrimination ability not required by the other tasks. Both tasks also required participants to respond to *infrequent* target trials, unlike the SART or MRT, which required frequent and consistent responding (with SART presenting visual stimuli and MRT auditory). Likewise, the Avengers AX-CPT and Motivation PVT modestly gamified traditional attention consistency measures to enhance engagement relative to the other tasks. We also included performance tasks that assessed accuracy (i.e., dʹ and omission rate), rather than just RT. With respect to self-report measures, we assessed TUTs via two different types of thought-content-focused probes across different tasks. As previously discussed—and as highlighted in Table 1 of Welhaf and Kane (2024b)—diverse sets of tasks and measures with different stimulus and response qualities allow researchers to account for variance in processes unrelated to sustained attention consistency that contribute to error construct measurement.

Some of our included measures were effective, while others were not. First, our “Avengers” version of the AX-CPT captured mostly variance unrelated to sustained attention consistency. The AX-CPT is typically used in studies to assess proactive cognitive control, which in principle might overlap to some degree with maintaining task readiness. It was only weakly correlated, however, with the other performance measures (and many of our participants were evidently confused about responding to both cue and target stimuli). Removing the AX-CPT from our models (along with SART omissions and MRT TUT rates; see below) in secondary analyses increased the correlation between performance and self-report latent factors and their loadings on a higher-order attention consistency factor. Proactive cognitive control may thus represent a related, but distinct form of attention from sustained attention consistency.

Second, TUT rate in the MRT may not be a reliable or robust indicator of sustained attention consistency. Although MRT TUT rate correlated with TUT rates from the other tasks, it did not correlate with the MRT performance measure and tended to correlate in the wrong direction with performance measures in the other tasks. Previous MRT studies have not always investigated between-person performance–TUT rate correlations; the few well-powered examples have reported weaker correlations than those typically elicited in other tasks, like the SART or PVT. For example, Anderson et al. (2021), Daniel et al. (2025) and Meier (2018), reported MRT performance–TUT rate correlations of *r* =.19,.06, and.18, respectively, with lower bounds of confidence intervals near zero. For comparison, correlations between SART performance and TUT rates typically range between *r* =.21–30 (e.g., Kane et al., 2016; McVay & Kane, 2012a; Welhaf & Kane, 2024b) and PVT performance and TUT rates between *r* =.26–40 (Robison & Unsworth, 2018; Unsworth & McMillan, 2014; Welhaf & Kane, 2024b).

It is also possible that variability in RT *and* speed of responding are important predictors in mind wandering in metronome-like tasks. For example, Javala and Wammes (2024) adopted the MRT to present trial-unique stimuli (e.g., pictures of rooms), which allowed them to test for later memory performance. During the task, participants periodically rated the depth of their mind wandering. They found that both variability and speed of responding prior to the thought probe predicted depth ratings. However, at the individual differences level, only RT variability was associated with depth of mind wandering (*r* =.38). For now, we hesitate to recommend using the MRT for individual differences studies of attention consistency until more construct validity work, with more reliable thought probe methods, is completed.

Overall, the task battery provided evidence for construct validity, which suggests that many of the included measures should continue to be used in future work on normal variation in sustained attention consistency. There are other tasks and measures that we did not include, but are also worth exploring. The gradCPT (Rosenberg et al., 2013) is a go/no-go task with a gradual transition between stimuli, rather than an abrupt switch, as in the SART. Recent work comparing abrupt versus gradual go/no-go tasks has shown that they measure similar processes. However, abrupt tasks also induce an element of (resisting) attention capture to the stimulus (Robison, 2023). Perhaps eliminating this contribution of capture could make the gradCPT (and other gradual presentation tasks) even more sensitive than the SART to fluctuations in attention consistency.

Recent work has also adapted the PVT into an accuracy-based measure known as the sustained attention-to-cue task, or SACT (Draheim et al., 2021, 2023). The SACT initially cues participants to a target location on the screen and then requires them to maintain attention over an unpredictable variable interval (from 2–12 s), much like the PVT. Following the delay interval, an array of letters is briefly presented and then masked. The participant’s goal is to identify the central letter correctly. Of importance here, SACT performance correlated strongly with PVT performance (*r* =.42; Draheim et al., 2021), suggesting they may be related but non-redundant measures of sustained attention consistency. The SACT also provides another avenue to assessing attention consistency using accuracy, rather than RT, which may benefit some task batteries. Future work should explore how strongly SACT performance correlates with the present task battery of attention consistency measures.

Regarding self-reports of attention lapses and mind-wandering, the probed TUT rate data analyzed in Welhaf and Kane (2024b) came from only one form of thought probe, which assessed prototypical thought-content categories via a forced-choice menu. The current study modestly broadened TUT assessment by probing thought content in two different ways, asking participants about either prototypical thought-content categories or the temporal orientation of their thought content. To our knowledge, no other studies have used multiple probe types across tasks, and within participants, to assess individual differences in TUT rates. Using different content-focused probe types in a single study may be useful, as different probe types may subtly affect participants’ experiences during the task and the way they report on their mind wandering (Kane et al., 2021a, 2021b; Robison et al., 2019; Weinstein, 2018). Relying on only a single content-based probe type in a study—as is typical—may increase measurement error related to TUT (and attention consistency) assessment. We acknowledge, though, that in the present study we could not model the two content-based probe types as providing distinguishable assessments of off-task thought rates; the two probe types yielded a single TUT rate factor, suggesting that they captured the same construct.

Likewise, among the performance measures, the current study could not distinguish accuracy-based from RT-based outcome factors. We have argued against relying on only one type of measurement approach for performance or self-reports because it increases measurement error (Welhaf & Kane, 2024b). The present findings, however, that accuracy and RT performance factors were indistinguishable, as were TUT rates from content-category versus temporal-content probes (as noted above), suggest that prior work on attention consistency (e.g., Kane et al., 2016; Unsworth et al., 2021; Welhaf & Kane, 2024b; Welhaf et al., 2020) was not significantly flawed in relying on one type of performance measure or thought probe.

Finally, future work on sustained attention consistency should consider the impact that gamification and motivational incentives have on altering the covariation between performance and self-report-based measures. Recent work has shown that manipulations of task parameters (e.g., task pacing and stimulus predictability) do not appear to alter the correlation between performance- and self-report-based attention consistency measures (Welhaf & Kane, 2024a). However, using more motivation-based manipulations together might be more powerful in changing this covariation between these measures. Recent work has used manipulations such as content-free cues and points (Unsworth et al., 2024), rewards and competition manipulations (Robison & Nguyen, 2023), specific goal-setting (Garner et al., 2025; Strayer et al., 2024), and manipulation of instructions to “try hard” (Unsworth et al., 2022a, 2022b), and even some combinations of the above (Robison et al., 2021) to alter mean levels of sustained attention performance and mind wandering. However, these studies have not explicitly examined how these manipulations might alter the individual differences covariation between performance- and self-report-based measures.

## Sustained attention consistency as the covariation in performance and self-report indicators

The correlations we found between performance and self-report latent variables were only of moderate strength (*r* =.29 and.37 in the initial and refined batteries, respectively, vs. *r*s =.32 and.38, in Welhaf & Kane, 2024b). Consistent with Welhaf and Kane (2024b), we suggest that the imperfect correlation between performance and thought-sampling indicators arises from each measurement type having their own substantial sources of error. That is, processes like speed–accuracy trade-offs, metacognitive expectation of task demands, or individual differences in error awareness (e.g., Polychroni et al., 2023; Rummel & Meiser, 2013; Xiao et al., 2015), could impact the measurement of performance-based attention consistency measures. In contrast, momentary self-reports of TUT experiences may be subject to different sources of error, such as individual capacities for meta-awareness, reporting biases, folk beliefs about mind wandering, or demand characteristics (e.g., Kane et al., 2021b; Vinski & Watter, 2012; Zedelius et al., 2015). As well, performance measures may be more sensitive to more subtle fluctuations in attention than are thought-sampling measures (i.e., those fluctuations not severe enough to generate subjective experiences of off-task thought).

We found that the shared individual-differences variance between these two measurement types was best modeled using a hierarchical approach, with a general attention consistency factor loaded by performance-specific and self-report-specific factors. As in Study 1 of Welhaf and Kane (2024b), the bifactor model, which simultaneously modeled a general sustained attention consistency factor along with orthogonal, measurement-specific factors, yielded an incoherent residual performance factor. Thus, we again provisionally suggest the hierarchical approach may be more pragmatic than the bifactor approach for assessing the general ability to sustain attention consistency and testing its associations with other constructs.

## Relations to conative factors provide additional construct validity evidence for attention consistency measurement

Engaging in sustained attention consistency appears to be an *ability* that individuals differ in. However, it is also possible that conative factors—like motivation and interest in the current task—affect how much, or how well, individuals engage sustained attention processes. Our previous work (Welhaf & Kane, 2024b) suggested that motivation and general attention consistency could not be modeled independently; that is, when both factors were included in the same structural model, the model did not adequately fit the data, and the correlation between the factors was estimated to be greater than 1.0. One of the current study's goals was to further assess the construct validity of our measurement approach for sustained attention consistency by examining its relations with conative factors, with those conative factors assessed with a valid multi-item (and multi-factor) instrument, completed before and after each task of interest.

Success motivation and task interest factors, derived from DSSQ motivation scale items (Matthews et al., 2002), both correlated as expected with the lower-order, measurement-specific attention consistency factors. Participants who endorsed caring more about task success and being more intrinsically interested in the task also reported fewer TUTs and had more consistent and accurate performance. However, results from the structural equation model, which tested for unique effects of each conative factor, suggested slightly different conclusions: Success motivation predicted unique variance in both measurement factors of sustained attention consistency, but task interest uniquely predicted only self-report measures. These findings contradict previously reported null associations between interest and mind wandering, even after accounting for motivation. For example, Unsworth and McMillan (2013) found that interest did not predict unique variance in TUT rates after accounting for motivation during passage reading. Likewise, He et al. (2023) showed that, despite moderate-to-strong latent correlations between task interest and motivation with TUT rate (*r*s = −.37 and −.53, respectively), the correlation between interest and TUT rate was fully mediated by motivation. It may be noteworthy that both latter studies assessed motivation and interest with only a one-item scale (He et al., 2023) or two-item scale (Unsworth & McMillan, 2013) for each construct, and only *after* completing the relevant tasks, thus potentially confounding the experience of attention lapses with subsequent reports of low motivation or interest.

As with our previous work (Welhaf & Kane, 2024b), the hierarchical model of sustained attention consistency indicated stronger relations with the conative factors than did the measurement-specific, two-factor model. Specifically, success motivation and task interest were strongly correlated with the higher-order attention factor (rs ≈..50), and the structural equation model indicated that both factors predicted unique variance in general sustained attention consistency. Thus, the individual differences covariation between performance-based and self-report-based factors of attention consistency provides a different pattern of results compared to a model using each factor on its own, with stronger correlations that are arguably more supportive of the construct validity of sustained attention consistency.

## Future recommendations for assessing sustained attention consistency

The current study attempted to broaden the assessment of sustained attention consistency by using different variations of performance-based measures (accuracy and RT) and different self-report approaches (content and temporal orientation thought probes). The results clearly suggested that within each domain, these different approaches were not separable, with correlations being.90–95. Future research might therefore benefit from using a mix of performance-based scores (i.e., both accuracy and RT based) and a mix of self-report-based scores (e.g., TUT rates from content, temporal orientation, emotional valence probes) to assess these constructs with optimal generality. As we argued earlier, using a variety of dependent variables can minimize the influence of non-attention-consistency factors, like speed–accuracy trade-offs or changes in response strategies for performance-based measures, and response biases to thought probes for self-report-based measures.

However, some tasks and measures from the current battery appeared to work better than others. For now, we recommend that individual-differences researchers use more validated sustained attention consistency measures such as the SART and PVT (both the standard and motivation versions) for measuring performance and self-report attention consistency within the same task. The CTET and DVG appear to work well for performance-based measures, but we do not yet know how well self-report measures within these tasks will work (see also the previously discussed gradCPT [Rosenberg et al., 2013] and SACT tasks [Draheim et al., 2021]). Finally, as previously discussed, the AX-CPT and MRT did not appear to be suitable for measuring individual differences in sustained attention consistency. While the AX-CPT is a well-validated measure of context processing and goal maintenance, the sustained attention consistency demand required by the task may not be great enough to drive individual variation in performance. Likewise, the MRT has produced reliable experimental time-on-task effects for both performance and TUTs reports (e.g., Anderson et al., 2021); Brosowsky et al., 2023; Laflamme et al., 2018) but correlations between MRT performance and TUT rates are inconsistent, suggesting less utility as an individual differences measure.

Additional measurement approaches could be added to task-performance and momentary-self-report indicators to further expand—and potentially improve—the psychometric assessment of normal variation in sustained attention consistency. Neurophysiological and neuroimaging signatures that covary with RT variability, performance lapses, or TUT reports (e.g., Kam et al., 2022; Machida et al., 2019; O’Connell et al., 2009; Ramchurn et al., 2014; Yamashita et al., 2021; Zuberer et al., 2021) are promising, but they may also be cost-prohibitive for large-N studies of individual differences.

Pupillometry, in contrast, may be a more realistic target for integration with performance and self-report measures in psychometric research. Pupil dilations may be indirectly related to locus coeruleus-norepinephrine system functioning (Aston-Jones et al., 1999; Aston-Jones & Cohen, 2005; but see Megemont et al., 2022), which is linked to physiological arousal and attention. Most relevant to our current operationalization of sustained attention consistency, fluctuations in pupillary responses may also (imperfectly) reflect momentary consistency of sustained attention (Hutchison et al., 2020; Unsworth & Robison, 2017a). Several studies have shown that lapses in attention, in the forms of performance indicators like RT variability and extremely slow RTs (e.g., Unsworth et al., 2018, 2020, 2022a, 2022b; Unsworth & Robison, 2016; Van Den Brink et al., 2016), and self-reported instances of mind wandering (e.g., Hutchison et al., 2020; Konishi et al., 2017; Pelagatti et al., 2018, 2020; Stawarczyk et al., 2020; Unsworth & Robison, 2018; Unsworth et al., 2022a, 2022b), are associated with changes in pupil size across many task contexts. Intrasubject variability in pupil size, in particular, may be a robust marker of individual differences in attention consistency (e.g., Robison & Brewer, 2022; Unsworth & Robison, 2017b).

Our fundamental argument is that future research should consider a *methodological triangulation* approach (Denzin, 1970) to assessing sustained attention consistency. Here, then, the individual differences covariation among performance variables, momentary self-reports of TUTs, and physiological indicators of attention consistency might optimally measure normal variation in the general ability to sustain attention consistency—if physiological indicators reflect construct-relevant variance that is not well captured by performance and thought-sampling measures.

## Conclusion

The ability to successfully maintain attention on a moment-to-moment basis is a fundamental cognitive process that has largely been understudied (Unsworth & Miller, 2021). The results of the current study support our previous work (Welhaf & Kane, 2024b), demonstrating that individual differences in sustained attention consistency ability may be best measured as the shared variance between task-performance and momentary-self-report indicators. This approach seems to provide a more construct valid measurement than either of these methods modeled individually. The current study offers insights into several important questions regarding measuring sustained attention consistency. First, different performance indicators (i.e., accuracy and RT variability) and different thought-sampling indicators (tapping different dimensions of off-task thoughts) do not appear to be distinguishable from one another. Second, conative factors (success motivation and task interest) are more strongly correlated with, and uniquely predict, the higher-order attention consistency factor than the task-performance or thought-sampling factors on their own.

## Electronic supplementary material

Below is the link to the electronic supplementary material.

Supplementary file1 (CSV 16 KB)

## Figures and Tables

**Fig. 9:**
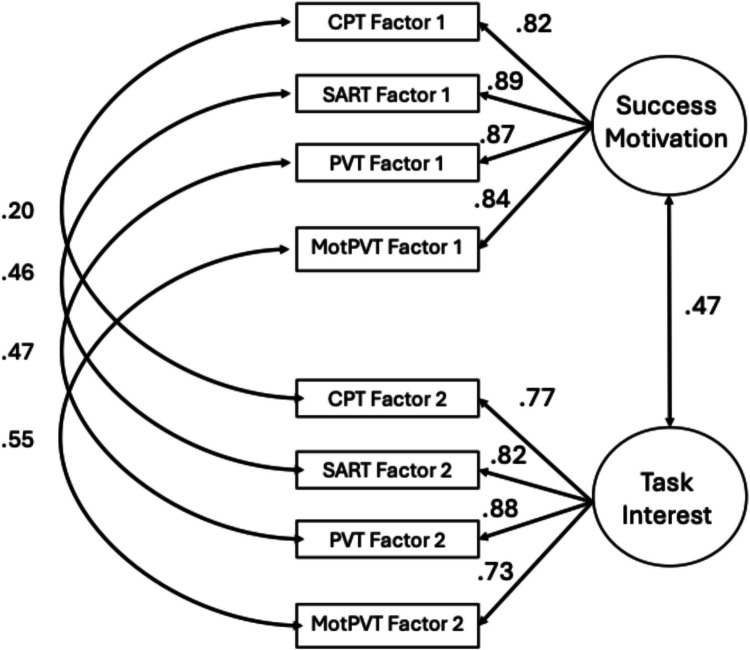
Measurement model of two-factor DSSQ

## Data Availability

Data and analysis scripts are available via the Open Science Framework (https://osf.io/jqtgb/?view_only=e10a9427aa4d4ebeb90986e47e061e98).

## Appendix

See Fig 9 and Table 7

**Table 7:** Factor loading table for refined models

Construct and measures	Model					
	Two-factor measurement	Hierarchical measurement	Two-factor CFA	Hierarchical CFA	Two-factor SEM	Hierarchical SEM
**Performance AC**						
SART RTSD	.61 (.04)	.61 (.04)	.61 (.04)	.61 (.04)	.61 (.04)	.61 (.04)
PVT Slowest 20%	.69 (.04)	.69 (.04)	.70 (.04)	.71 (.04)	.70 (.04)	.71 (.04)
MRT RRT	.60 (.04)	.60 (.04)	.59 (.04)	.59 (.04)	.59 (.04)	.59 (.04)
MOT PVT Slowest 20%	.57 (.05)	.57 (.05)	.59 (.05)	.59 (.05)	.59 (.05)	.59 (.05)
DVG d’	– .53 (.04)	– .53 (.04)	– .51 (.04)	– .51 (.04)	– .51 (.04)	– .51 (.04)
CTET d’	– .70 (.03)	– .70 (.03)	– .70 (.03)	– .70 (.03)	– .70 (.03)	– .70 (.03)
**Self-Report AC**						
SART TUTs	.60 (.05)	.60 (.05)	.61 (.05)	.60 (.05)	.61 (.05)	.60 (.05)
PVT TUTS	.77 (.05)	.77 (.05)	.75 (.05)	.76 (.05)	.75 (.05)	.76 (.05)
MOT PVT TUTS	.54 (.05)	.51 (.05)	.55 (.05)	.54 (.05)	.54 (.05)	.54 (.05)
**Motivation Factor 1**						
AX-CPT Factor 1			.82 (.03)	.82 (.03)	.82 (.03)	.82 (.03)
SART Factor 1			.89 (.02)	.89 (.02)	.89 (.02)	.89 (.02)
PVT Factor 1			.88 (.02)	.88 (.02)	.88 (.02)	.88 (.02)
MOT PVT Factor 1			.84 (.02)	.84 (.02)	.84 (.02)	.84 (.02)
**Motivation Factor 2**						
AX-CPT Factor 2			.76 (.03)	.76 (.03)	.76 (.03)	.76 (.03)
SART Factor 2			.82 (.02)	.81 (.02)	.82 (.02)	.81 (.02)
PVT Factor 2			.89 (.02)	.89 (.02)	.89 (.02)	.89 (.02)
MOT PVT Factor 2			.73 (.03)	.73 (.03)	.73 (.03)	.73 (.03)

SART = Sustained Attention to Response Task; PVT = Psychomotor Vigilance Task; Mot = Motivation; MRT = Metronome Response Task; RRT = Rhythmic Response Time; DVG = Degraded Vigilance Task; CTET = Continuous Temporal Expectancy Task; TUT = Rate of Task-Unrelated Thoughts.

## Footnotes

1 Participants also completed a Finger Tapping task (after the SART) which required them to maintain a briefly practiced rhythm after the withdrawal of the guiding rhythmic auditory stimuli. Compared to the auditory MRT, this task was more reliant on endogenous attentional consistency because there was no overt stimulus that triggered each response. However, this task had severe distributional issues (even after attempts at correction) and did not correlate with other performance attention consistency measures. Thus, we did not analyze data from this task further.

2 Our decision to use this outlier procedure was guided by previous mini-multiverse analyses, which tested how different trial- and subject-level outlier decisions impacted latent-variable correlations (e.g., Welhaf & Kane, 2024b; Welhaf et al., 2020). By censoring outlying trials to a subject-specific cutoff, we can retain all available data without the undue influence of outlying trials (which could be caused by processes unrelated to attention consistency, such as blinking or sneezing). In both cases, outlier decisions had little impact on the correlations. Most important for the current study, our reanalysis in Welhaf & Kane (2024b) showed that different outlier decisions did not impact the fit or structure of the hierarchical sustained attention consistency model.

3 It is worth noting that in several of the initial models, TLI was just below the recommended cutoff of.90 (typically.89), but because this was the only fit indicator below threshold throughout, and because no modification indices suggested model changes that would have been justifiable a priori, we chose to keep the models as-is.

4 We also fit two 3-factor models (combining the two performance factors or combining the two TUT-rate factors). Again, the data fit these models well (see Table 1), but a similar warning suggested the two performance factors and two TUT-rate factors were not separable.

5 We also considered a one-factor model, in which all attention consistency indicators loaded onto a single factor. This model did not adequately fit the data (see Table 1).

6 By dropping these seemingly poor indicators, we also noticed that TLI increased from just under.90 to above.90 and so all model fit indices were above our a priori threshold.

## Declarations

### Open practice statement

Data and analysis scripts are available via the Open Science Framework (https://osf.io/jqtgb/?view_only=e10a9427aa4d4ebeb90986e47e061e98). This study was not preregistered.
